# Comparison and development of machine learning tools for the prediction of chronic obstructive pulmonary disease in the Chinese population

**DOI:** 10.1186/s12967-020-02312-0

**Published:** 2020-03-31

**Authors:** Xia Ma, Yanping Wu, Ling Zhang, Weilan Yuan, Li Yan, Sha Fan, Yunzhi Lian, Xia Zhu, Junhui Gao, Jiangman Zhao, Ping Zhang, Hui Tang, Weihua Jia

**Affiliations:** 1 Department of Pulmonary and Critical Care Medicine, General Hospital of Datong Coal Mine Group Co., Ltd., Datong, 037000 China; 2 Department of Respiratory, General Hospital of Tisco (Sixth Hospital of Shanxi Medical University), 2 Yingxin Street, Jiancaoping District, Taiyuan, 030008 Shanxi Province China; 3 Department of Respiratory, Linfen People’s Hospital, Linfen, 041000 China; 4 Shanghai Biotecan Pharmaceuticals Co., Ltd., 180 Zhangheng Road, Shanghai, 201204 China; 5 Shanghai Zhangjiang Institute of Medical Innovation, Shanghai, 201204 China; 6 Department of Respiratory Medicine, Hebei General Hospital, Shijiazhuang, 050000 China; 7 Department of Respiratory Medicine, Heji Hospital Affiliated with Changzhi Medical College, Changzhi, 046011 China; 8 Department of Clinical Laboratory, JinCheng People’s Hospital, Jincheng, 048000 China; 9 Department of Clinical Laboratory, Linfen People’s Hospital, West of Rainbow Bridge, West Binhe Road, Yaodu District, Linfen, 041000 Shanxi Province China; 10 Department of Pulmonary and Critical Care Medicine, The First Hospital of Shanxi Medical University, Taiyuan, 030001 China

**Keywords:** COPD, SNP, AQCI, Allele frequencies, Machine learning tools

## Abstract

**Background:**

Chronic obstructive pulmonary disease (COPD) is a major public health problem and cause of mortality worldwide. However, COPD in the early stage is usually not recognized and diagnosed. It is necessary to establish a risk model to predict COPD development.

**Methods:**

A total of 441 COPD patients and 192 control subjects were recruited, and 101 single-nucleotide polymorphisms (SNPs) were determined using the MassArray assay. With 5 clinical features as well as SNPs, 6 predictive models were established and evaluated in the training set and test set by the confusion matrix AU-ROC, AU-PRC, sensitivity (recall), specificity, accuracy, F1 score, MCC, PPV (precision) and NPV. The selected features were ranked.

**Results:**

Nine SNPs were significantly associated with COPD. Among them, 6 SNPs (rs1007052, OR = 1.671, *P *= 0.010; rs2910164, OR = 1.416, *P *< 0.037; rs473892, OR = 1.473, *P *< 0.044; rs161976, OR = 1.594, *P *< 0.044; rs159497, OR = 1.445, *P *< 0.045; and rs9296092, OR = 1.832, *P *< 0.045) were risk factors for COPD, while 3 SNPs (rs8192288, OR = 0.593, *P *< 0.015; rs20541, OR = 0.669, *P *< 0.018; and rs12922394, OR = 0.651, *P *< 0.022) were protective factors for COPD development. In the training set, KNN, LR, SVM, DT and XGboost obtained AU-ROC values above 0.82 and AU-PRC values above 0.92. Among these models, XGboost obtained the highest AU-ROC (0.94), AU-PRC (0.97), accuracy (0.91), precision (0.95), F1 score (0.94), MCC (0.77) and specificity (0.85), while MLP obtained the highest sensitivity (recall) (0.99) and NPV (0.87). In the validation set, KNN, LR and XGboost obtained AU-ROC and AU-PRC values above 0.80 and 0.85, respectively. KNN had the highest precision (0.82), both KNN and LR obtained the same highest accuracy (0.81), and KNN and LR had the same highest F1 score (0.86). Both DT and MLP obtained sensitivity (recall) and NPV values above 0.94 and 0.84, respectively. In the feature importance analyses, we identified that AQCI, age, and BMI had the greatest impact on the predictive abilities of the models, while SNPs, sex and smoking were less important.

**Conclusions:**

The KNN, LR and XGboost models showed excellent overall predictive power, and the use of machine learning tools combining both clinical and SNP features was suitable for predicting the risk of COPD development.

## Background

It has been reported that chronic obstructive pulmonary disease (COPD) is a public health challenge due to its high prevalence and related disability, mortality and socioeconomic burden worldwide [1–3]. Approximately 90% of deaths related to COPD occur in Asia and Africa [4]. In 2013, more than 0.9 million deaths related to COPD occurred, and COPD was reported to be the third leading cause of death in China [5].

The typical symptoms of COPD include dyspnea, chronic cough, and sputum production, and spirometry is considered the gold-standard method for the diagnosis of COPD [6]. Spirometry is essential for diagnosis and provides a useful description of the severity of pathologic changes in COPD. The forced expiratory volume in one second (FEV1), forced vital capacity (FVC), and the ratio of FEV1 to FVC are used to evaluate pulmonary function [7]. COPD is now clinically defined as a post-bronchodilator FEV1/FVC less than 70% of the predicted value and FEV1 less than 80% of the predicted value [8].

Rehman et al. [3] reported that in Europe and the USA, the prevalence of COPD ranges from 3.4 to 13.4%, whereas in Asia, the prevalence ranges from 3.5 to 19.1% [9] due to urbanization, industrial pollution, tanneries and the sue of biomass fuel inside homes [10].

Smoking is a well-known risk factor for COPD development; however, fewer than 20% of smokers develop COPD, and more than 15% of non-smokers have COPD [11]. Recent studies have shown that many people develop COPD without ever smoking. Therefore, other factors besides personal smoking, other environmental triggers, such as second-hand smoke during pregnancy or early childhood, various genetic factors, occupational exposure to dust, noxious fumes and vapors, indoor air pollution from the use of biomass fuels, and outdoor air pollution, may interact in an additive manner with risk factors within individual and lifestyle issues (diet and exercise) [12]. Zhong reported that COPD was more common among rural residents than among urban residents in China, probably because of a number of environmental and individual risk factors, such as old age, smoking, coal use, infection, and low body mass index (BMI) [13]. In addition, it has been reported that infections could promote the progression of COPD, such as in patients with emphysema and adenoviral infections or patients with asthma and intracellular infections [14–16]. Beyer et al. reported that COPD may originate in childhood or even in utero. Lung function can be compromised during lung development in utero—e.g., low birthweight babies or children whose mothers smoked during pregnancy have reduced lung function soon after birth [17].

Wang et al. reported a national cross-sectional study in China that indicated that COPD was highly prevalent in the Chinese adult population. Cigarette smoking, ambient air pollution, underweight, childhood chronic cough, parental history of respiratory diseases, and low education are major risk factors for COPD in the Chinese population. Among these factors, cigarette smoking and air pollution are major preventable risk factors for this disease [18]. With rapid industrialization and urbanization, ambient air pollution has become a major public health crisis in China [19]. The air quality composite index (AQCI) was obtained from the Chinese Official website, which included the pollution degree of the six pollutants SO_2_, NO_2_, PM_10_, PM_2.5_, CO and O_3_ and other pollutants. The higher the AQCI is, the more serious the pollution. In the present study, we used a combination of AQCI and other risk factors, such as age, sex, BMI and smoking, to predict COPD development.

In addition, COPD susceptibility- or disease progression-related genes have been reported [20], and genome-wide association studies (GWASs) have revealed single nucleotide polymorphism (SNP) sites related to COPD occurrence and development, such as *HHIP* [21], *IL13* [22], *MMP9* [23], *SFTPB* [24], *SOD3* [25], *CHRNA3* [26], *RNF150* [27], BICD1 [28], *COL4A3* [29], *AQP5* [30], *AGPHD1* [31], *IREB2* [32], etc. Many studies have indicated that some candidate genes are associated with COPD over the past few years. However, there have been few studies on the susceptible loci of COPD in the Chinese population in recent years (Additional file 1: Table S1).

Lung function is the gold standard for the clinical diagnosis of COPD; however, when the FEV1/FVC or FEV1% value is abnormal, lung function is a defective indicator in approximately 30% of patients [33]. COPD in the early stages is usually not recognized, diagnosed, or treated and therefore may not be included as a diagnosis in patient medical records. Therefore, the applicability of the lung function index for the early diagnosis of COPD is limited. With the rapid aging of the Chinese population, COPD has become one of the leading causes of disability and a large economic burden [34]. Therefore, it is necessary to develop a reliable early warning method for COPD that could lead to early intervention and treatment for COPD.

In the present study, we performed a case–control study including 441 patients with COPD and 192 healthy controls. Then, the odds ratios (ORs) of the genotypes of 101 SNPs and clinical features for COPD development were calculated. We established and compared six prediction models that included susceptible SNPs and clinical features using statistical, machine learning and neural network approaches.

## Methods and materials

### Study population

A total of 441 COPD patients and 192 control subjects were randomly recruited from seven subcenters in China from January to December 2017, including Linfen People’s Hospital (Linfen city, Shanxi Province), Jincheng People’s Hospital (Jincheng city, Shanxi Province), Heji Hospital Affiliated with Changzhi Medical College (Changzhi city, Shanxi Province), General Hospital of Tisco (Sixth Hospital of Shanxi Medical University) (Taiyuan city, Shanxi Province), Hebei General Hospital (Shijiazhuang city, Hebei Province), General Hospital of Datong Coal Mine Group Co., Ltd. (Datong city, Shanxi Province), and Shanghai Zhangjiang Institute of Medical Innovation (Shanghai). COPD was diagnosed according to the Global Initiative for Chronic Obstructive Lung Disease (GOLD) criteria [6], and patients with other medical histories were excluded. All procedures were performed in accordance with the ethical standards of the Clinical Research Ethics Committee of the above hospitals, and informed consent was obtained from all individuals included in the study.

The basic characteristics of all participants are listed in Additional file 2: Tables S2 and Additional file 3: Table S3. Smoking status was defined as follows: nonsmokers had never smoked, and smokers included ex-smokers and current smokers. BMI was measured in kg/m^2^(underweight < 19, normal = 19–25, overweight = 25–30, obese ≥ 30). AQCI values were derived from the following seven regions according to official government website statistics in China: five cities from Shanxi Province (http://sthjt.shanxi.gov.cn/html/tndt/20180119/58694.html), Shijiazhuang from Hebei Province (http://hbepb.hebei.gov.cn/hjzlzkgb/) and Shanghai (http://www.mee.gov.cn/hjzl/zghjzkgb/lnzghjzkgb/).

### DNA extraction and genotyping

We selected 101 SNPs from 76 genes and 9 intergenic regions that were previously reported to be associated with COPD [20, 23–29, 32, 35–60]. A 4-mL peripheral blood sample was obtained from each participant for DNA analysis. Genomic DNA was extracted from whole blood using the GoldMag-Mini Whole Blood Genomic DNA Purification Kit (GoldMag Co. Ltd., Xi’an City, China). The DNA concentration was measured using a NanoDrop 2000 (Thermo Scientific, Fitchburg, WI, USA).

### MassArray assay

We used the https://agenacx.com website to design multiplex primers for each SNP: 1st PCR primer, 2nd PCR primer, and UEP primer. The primers for the 101 SNPs are shown in Additional file 4: Table S4. The SNPs were genotyped with an Agena BioscienceTMMassARRAY^®^ Analyzer 384-well Configuration (Agena, CA, USA) according to the standard protocol recommended by the manufacturer.

PCR amplification was performed in a reaction system with a total volume of 5 μl containing 10 ng genomic DNA, 1 U PCR enzyme (Agena), 0.5 μl 10× PCR buffer, 0.1 μl dNTPs mix and 0.5 μl of each primer under the following program: 2 min denaturation at 95 °C, 45 cycles of 30 s at 95 °C, 30 s at 56 °C and 60 s at 72 °C and a final extension at 72 °C for 5 min. Then, the PCR products were cleaned by 2 μl SAP (Agena) including 1.53 μl nanopure water, 0.17 μl SAP buffer and 0.5 U SAP enzyme with the following steps: 40 min at 37 °C and 5 min at 85 °C. Finally, the single-base extension used 2 μl iPLEX EXTEND mix (Agena) containing 0.619 μl nanopure water, 0.94 μl Extend primer mix, 0.041 μl iPLEX enzyme, 0.2 μl iPLEX buffer and 0.2 μl iPLEX termination mix and was performed with the following steps: initial denaturation at 94 °C for 30 s, followed by 40 cycles of a 3-step amplification profile of 5 s at 94 °C, an additional 5 cycles of 5 s at 52 °C and 5 s at 80 °C and a final extension at 72 °C for 3 min. Data management and analysis were performed using Typer Analyzer 4.0 software (Agena). Several SNP samples were finally excluded because ≥ 10% of the genotyping data were missing.

### Model construction in the training set

First, 290 COPD patients and 103 control subjects were enrolled as the training set, and the OR values of all genotypes of SNPs were calculated using the PLINK software package (version 1.07) [61]. The genotypes with missing OR values were assigned the average OR value. Nine SNPs were identified to be significantly associated with COPD risk in the Chinese population. Then, 6 models were established to predict COPD development, including a logistic regression (LR) model, an artificial neural network of the multilayer perceptron (MLP), a decision tree model (DT), a XGboost model, a support vector machine (SVM) and a k-nearest neighbors classifier (KNN) model, and included 5 clinical features and 9 SNPs. K-folder cross validation (k = 5) was used to train, construct and compare the 6 predictive models. The confusion matrix, area under the receiver operating characteristic (ROC) curve (AU-ROC), the area under the precision-recall (PR) curve (AU-PRC), specificity, sensitivity (recall), positive predictive value (PPV (precision)), negative predictive value (NPV), accuracy, F1 score and MCC were used to evaluate and compare the comprehensive performance of feature selection. AU-ROC is one of the most used metrics in evaluating binary classifiers and shows the true positive rate against the false positive rate. Compared with AU-ROC, AU-PRC is useful for unbalanced data, such as our study, and shows precision against recall. The F1 score takes the harmonic mean of precision and recall [62]. The MCC result ranges between -1 and 1, where a value of 1 indicates a perfect positive correlation, a value of -1 indicates a perfect negative correlation, and a value of 0 indicates no correlation [63].

Model selection was based on several currently and frequently adopted predictive model types. For example, the linear LR model [64] and SVM model have been widely adopted in many clinical applications, such as for CKD disease prediction [65]. The DT model [66] is based on a radial basis function neural network and support vector machine coupled with firefly algorithm techniques; the XGboost and MLP models have also been used in clinical research [65, 67]. KNN was chosen due to its simplicity and ability to perform multiclass classification, and this algorithm could run with default parameters [68]. When tuning the parameters in the KNN, SVM and DT models, the overall effect did not perform as well as choosing default parameters, so tuning parameters were not chosen in the three models, while tuning parameters were used in the LR, MLP and XGboost models. All the corresponding parameters are listed in Additional file 5: Table S5.

### Assessment of the six models in the test set

**Fig. 1:**
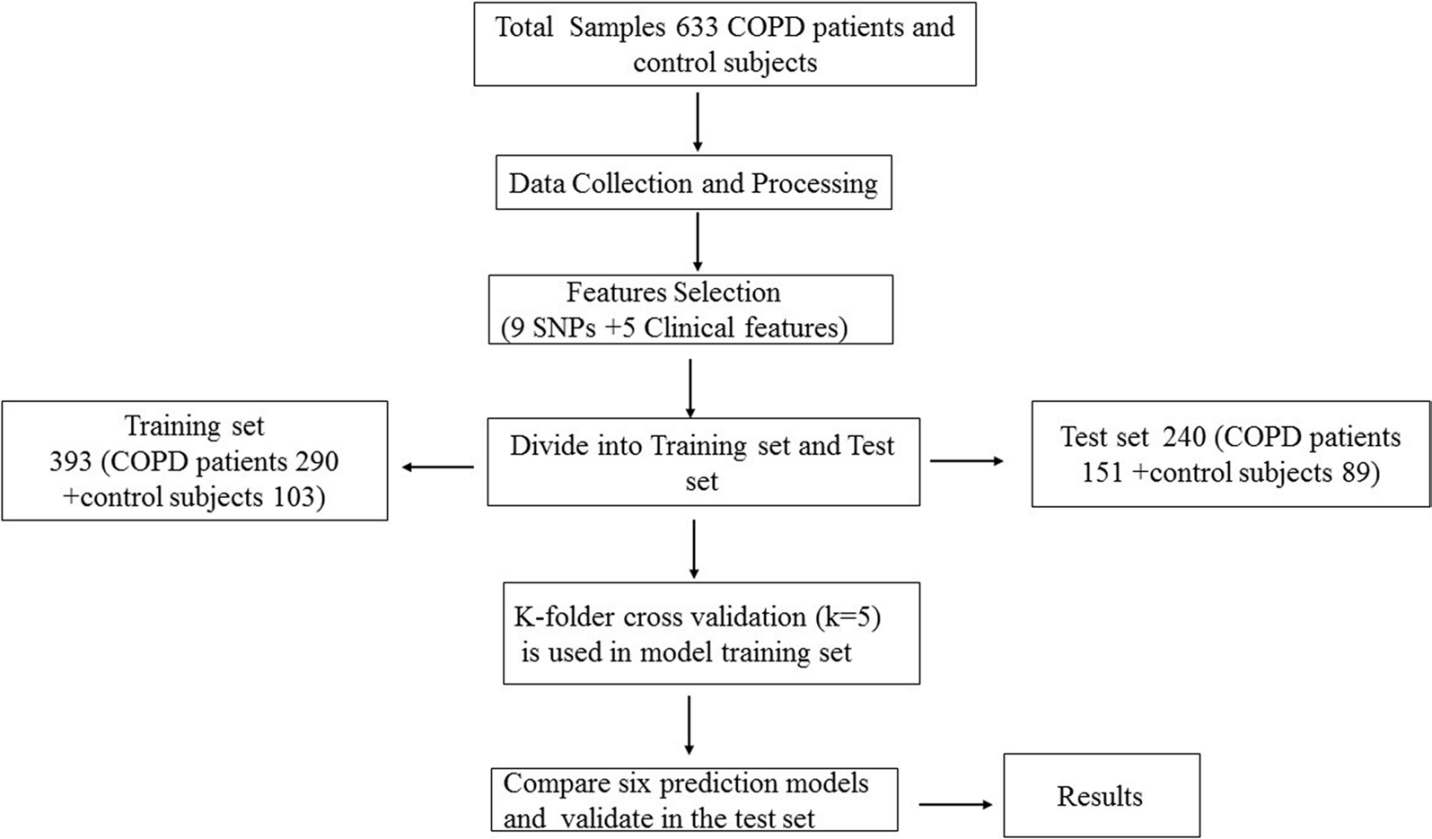
Flow chart display. Flow chart showing the SNP selection, model training, and performance evaluation processes. A total of 633 subjects were recruited for the current study. The data were preprocessed and randomly divided into a training set (393 participants) and a test set (240 participants). k-fold cross-validation was used in the training set, and performance evaluation indexes such as AU-ROC and AU-PRC were adopted to judge the average predictive performance of each model

In the validation set, we no longer calculated the OR values from PLINK software and directly mapped the OR value of each genotype from the training set. However, the genotypes with missing OR values were assigned the average OR value independently in the test set. To validate the training set, we recruited 151 COPD patients and 89 controls in the test set. Six models were selected for validation in the test set. The entire process is shown in Fig. 1. All the input data and sample output folders were uploaded to GitHub (https://github.com/weilan-yuan/COPD_machine-learning).

### Statistical analysis

For all SNPs, the ORs and 95% confidence intervals (CIs) of the minor alleles were assessed without adjusting for age, sex, BMI, smoking status and AQCI by Chi squared tests using the PLINK package between COPD patients and healthy controls. Furthermore, six predictive models (KNN, LR, DT, SVM, XGboost and MLP) for COPD risk were used and evaluated by Python (version 3.7.0) and included 9 SNPs with 5 clinical features. The diagnostic values of the 6 models were assessed by ROC and PRC analysis. Parametric statistics (*t*-test) were used for normally distributed data, and nonparametric statistics (Mann–Whitney) were used for non-normally distributed data. The *t*-test and nonparametric Mann–Whitney U test or Chi squared test was used to compare parametric and categorical variables, respectively. Statistical calculations were performed in R studio (R.3.51). *P *≤ 0.05 was the threshold for statistical significance.

## Results

### Clinical characteristics of the participants

The clinical characteristics of the cases and controls are shown in Additional file 2: Tables S2 and Additional file 3: Table S3. There were significant differences in age, sex, smoking status, AQCI, FEV1/FVC (%) and FEV1 (%) between COPD patients and healthy controls (*P *< 0.0001) in both the training and test sets. Only the BMI levels were similar between the two groups. The results indicated that COPD patients were more likely to be older, male, and smokers, and the FEV1/FVC (%) and FEV1 (%) values were lower in the COPD group than in the healthy controls.

### Allele frequency comparisons between the two groups

**Fig. 2:**
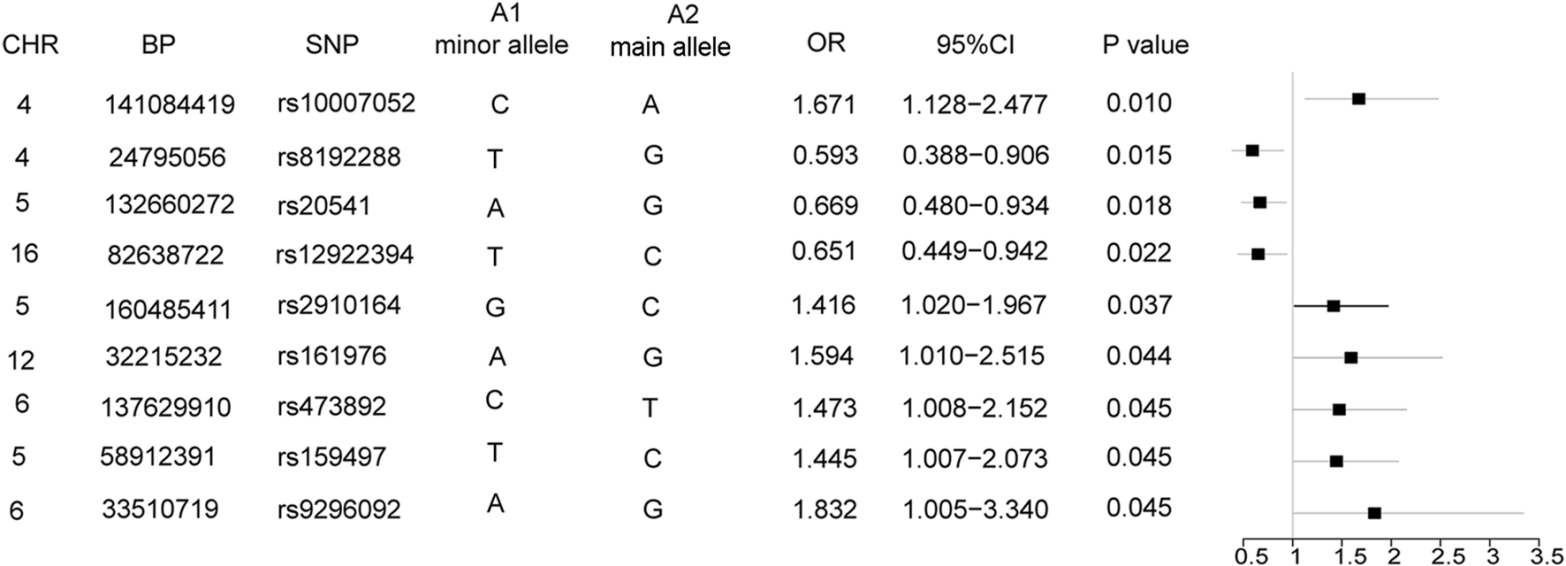
Nine SNPs associated with COPD. Forest plots show 9 SNPs associated with COPD (odds ratios (ORs) and 95% confidence intervals (CIs)). ORs are denoted by black boxes, and 95% CIs are denoted by the corresponding black lines

In the training set, 6 SNPs (rs3025030, rs28929474, rs7326277, rs7326277, rs16969968, and rs59569785) were excluded because ≥ 10% of the sample data were missing. Finally, 95 of the 101 SNPs were included in the following analysis. We assumed that the minor allele (A1) of each SNP was a risk factor compared to the main allele (A2) and obtained the OR values. All results are shown in Additional file 6: Table S6. The results showed that 9 SNPs tended to be significantly associated with COPD: 6 SNPs (rs1007052, rs2910164, rs159497, rs473892, rs9296092 and rs161976) were risk factors for COPD development, while 3 SNPs (rs8192288, rs12922394 and rs20541) were protective factors against developing COPD, as shown in the forest plots in Fig. 2.

### The role of six risk SNPs in COPD

Among the 6 risk SNPs, the SNP rs10007052 is located in the first intron of the *RNF150* gene. Ding et al. first reported that polymorphisms of *RNF150* were significantly associated with COPD risk in the Chinese Han and Li populations [27]. In our present case–control study, we also found that the SNP rs10007052 affected the pathogenesis of COPD in a Chinese population (rs1007052, OR = 1.671, 95% CI 1.128–2.477, *P *= 0.010). rs2910164 was reported as a C/G polymorphism in the precursor stem region of pre-miR146a on chromosome 5q33 and was first associated with genetic predisposition towards papillary thyroid cancer [69]. In the current study, we found that the G allele of the rs2910164 SNP (OR = 1.416, 95% CI 1.020–1.967, p < 0.037) is a risk factor for COPD development. Regarding rs473892, which is located in intergenic regions at chromosome 6 near the gene *OLIG3,* the T allele of rs473892 was associated with a higher FEV1 level in subjects with high exposure to mineral dust than in those without exposure in the LifeLines and Vlagtwedde-Vlaardingen samples [70]. In the present study, we found that the C allele of rs473892 (OR = 1.473, 95% CI 1.008–2.152, *P *< 0.045) was a risk factor for COPD development. These findings indicated that the T allele of rs473892 may be a protective locus for COPD, while the C allele is a risk locus for COPD. It has been reported that *BICD1 (*rs161976) is a potential susceptibility gene in COPD patients. rs161976 was previously demonstrated in a GWAS to be associated with emphysema in patients with COPD with an FEV1 < 80% of the predicted value [71]. In the present study, we found that rs161976 (OR = 1.594, 95% CI 1.010–2.515, *P *< 0.044) is also a risk locus in COPD. rs159497 is located in intergenic regions near the *PDE4D* gene. It was reported that *PDE4D* was not only a susceptibility gene for asthma [72] but also for ever-smokers who were associated with a reduced FEV1 level [73]. In the current study, we also showed that rs159497 (OR = 1.445, 95% CI 1.007–2.073, *P *< 0.045) was a risk factor for COPD development. rs9296092 is located in intergenic regions that lie in the gene region between the zinc finger and BTB domain containing 9 and BCL2-antagonist/killer1 (*ZBTB9*-*BAK1*) at chromosome 6 p21.32. Ding et al. also reported that in a case–control study, rs9296092 was associated with the greatest increase in the risk of COPD in the Hainan Province of China [74]. In our current study, we found that the A allele of rs9296092 (OR = 1.832, 95% CI 1.005–3.34, *P *< 0.045) is also a risk factor for COPD development.

### The roles of rs8192288, rs20541 and rs12922394 in COPD

In addition, 3 SNPs were protective against COPD; among them, the SNP rs8192288 is located in the first intron of the *SOD3* gene [25]. In our present results, we found that the T allele of rs8192288 (OR = 0.593, 95% CI 0.388–0.906, *P *< 0.015) was associated with a reduced risk of COPD. The SNP rs20541 is in the fourth exon of the *IL*-*13* gene [75]. In the present study, we found that the A allele of rs20541 (OR = 0.669, 95% CI 0.480–0.942, *P *< 0.018) is a protective factor against COPD. The SNP rs12922394 is in the first intron of the *CDH13* gene and is usually removed during the gene-splicing process. Yuan et al. reported that the T allele of rs12922394 was associated with a significantly reduced risk of COPD [44]. In the current study, we found that the T allele of rs12922394 (R = 0.651, 95% CI 0.449–0.942, *P *< 0.022) was also associated with a reduced risk of COPD, indicating that this variant may protect against COPD development.

### Comparison of 6 prediction models in the training set

**Fig. 3:**
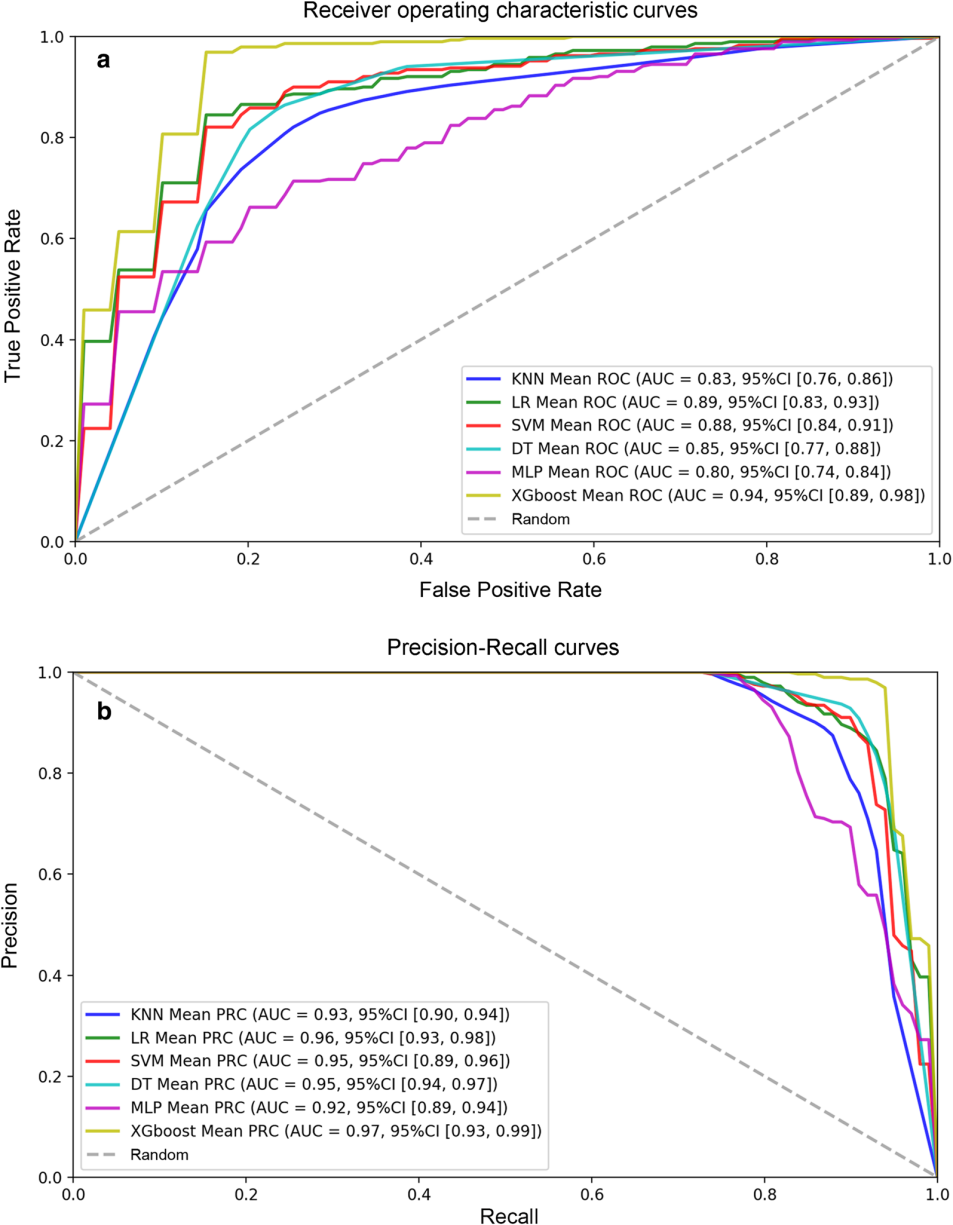
Evaluation of the predictive models. **a**, **b** The picture shows the AU-ROC and AU-PRC curves of the 6 models in the training set. Mean AUC values and 95% CIs of different prediction models are shown in the box

**Fig. 4:**
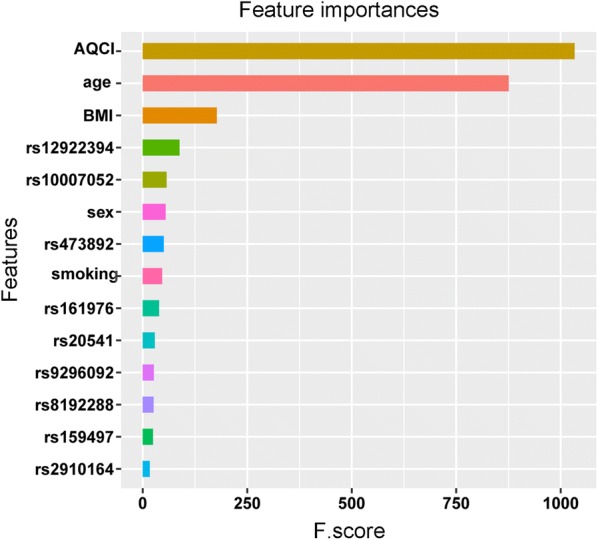
Analysis of the importance of each feature. The histogram describes the relative importance of 9 SNPs and 5 clinical features in the XGboost model. The relative importance is quantified by assigning a weight between 0 and 1000 for each variable

In the training set, k-fold cross-validation (k = 5) was used, and various parameter combinations were exhausted by grid search. Six models were established in this study. For each model, the evaluation indicators used were the confusion matrix, AU-ROC, AU-PRC, specificity, sensitivity (recall), PPV (precision), NPV, accuracy, F1 and MCC score. The average AU-ROC, AU-PRC and 95% CI are shown in Fig. 3a, b. Five models had AU-ROC values above 0.82, and only the MLP model had a lower value (0.80). The AU-PRC values were above 0.91 for all models. Otherwise, the six models presented varying performances, as shown in Table 1. Five models, namely, KNN, LR, SVM, DT and XGboost, had excellent performance, and the accuracy, PPV (precision), sensitivity (recall), and F1 score were above 0.81, 0.85, 0.87 and 0.87, respectively. Among the five models, the MCC, specificity and NPV were above 0.69, 0.78 and 0.80, respectively, for both the DT and XGboost models; the MLP model obtained the highest sensitivity (recall) (0.99) and NPV (0.87) but had the lowest specificity rate (0.10). XGboost obtained the highest AU-ROC value of 0.94 (95% CI 0.89–0.98) and AU-PRC value of 0.97 (95% CI 0.93–0.99), with the highest accuracy (0.91), PPV (precision) (0.95), F1 score (0.94), MCC (0.77) and specificity (0.85). The results indicated that XGboost was the best-performing model in the training set. Therefore, we used the XGboost model to analyze the importance of features including 9 SNPs and 5 clinical features, and the feature score (F. score) rankings were measured by the total_gain metric in XGboost (Fig. 4). The results showed that location (AQCI), age and BMI played important roles in the model, while 9 SNPs, smoking status and sex were less important.

**Table 1:** The efficacy of KNN, LR, SVM, DT, MLP and XGboost in the training set

Metrics	KNN	LR	SVM	DT	MLP	XGboost
	(95% CI)	(95% CI)	(95% CI)	(95% CI)	(95% CI)	(95% CI)
AU-ROC	0.83 (0.76–0.86)	0.89 (0.83–0.93)	0.88 (0.84–0.91)	0.85 (0.77–0.88)	0.80 (0.74–0.84)	0.94 (0.89–0.98)
AU-PRC	0.93 (0.90–0.94)	0.96 (0.93–0.98)	0.95 (0.89–0.96)	0.95 (0.94–0.97)	0.92 (0.89–0.94)	0.97 (0.93–0.99)
Accuracy	0.82 (0.77–0.86)	0.83 (0.77–0.86)	0.84 (0.82–0.88)	0.89 (0.84–0.92)	0.76 (0.74–0.79)	0.91 (0.88–0.95)
Precision	0.88 (0.83–0.92)	0.86 (0.83–0.89)	0.88 (0.84–0.91)	0.92 (0.89–0.95)	0.76 (0.74–0.79)	0.95 (0.93–0.96)
Recall	0.88 (0.85–0.90)	0.91 (0.85–0.96)	0.92 (0.90–0.95)	0.94 (0.92–0.98)	0.99 (0.98–1.00)	0.93 (0.88–0.97)
F1 score	0.88 (0.84–0.90)	0.89 (0.84–0.91)	0.90 (0.88–0.92)	0.93 (0.91–0.96)	0.86 (0.85–0.88)	0.94 (0.91–0.96)
MCC	0.54 (0.39–0.64)	0.54 (0.39–0.61)	0.58 (0.51–0.69)	0.70 (0.57–0.79)	0.22 (0.10–0.40)	0.77 (0.70–0.86)
SPC	0.67 (0.53–0.80)	0.59 (0.48–0.71)	0.63 (0.50–0.76)	0.79 (0.72–0.85)	0.10 (0.05–0.26)	0.85 (0.81–0.90)
NPV	0.66 (0.56–0.71)	0.72 (0.56–0.83)	0.74 (0.69–0.79)	0.81 (0.70–0.98)	0.87 (0.54–1.00)	0.81 (0.73–0.89)

*AU-ROC* area under the receiver operating characteristic curve, *AU-PRC* area under the precision-recall curve, *MCC* Matthews correlation coefficient, *SPC* specificity, *NPV* negative prognostic value, *KNN* k-nearest neighbors classifier, *LR* logistic regression, *SVM* support vector machine, *DT* decision tree, *MLP* multilayer perceptron, 95% CI 95% confidence interval

To verify the importance of clinical features or SNPs for predicting COPD in all models, we used only 9 SNPs and 5 clinical features and selected the top 5 ranked features, which included 3 clinical features (AQCI, age and BMI) and 2 SNPs (rs12922394 and rs10007052), to be included in the six models. The results indicated that with only the input of 9 SNPs, the AU-ROC values were only 0.51–0.66, but the AU-PRC values of all models were 0.80–0.86 (Additional file 7: Fig. S1). The PPV (precision), sensitivity (recall) and F1 score of all models were above 0.70, and the accuracy rate was above 0.61. However, the other evaluation indexes, such as MCC, specificity and NPV, were poor for all six models (Additional file 8: Table S7). The AU-PRC is an alternative approach for assessing the performance of a biomarker and is a summary statistic that reflects the ability of a biomarker to identify the diseased group [76]. In the present study, we found that with only the input of the 9 SNPs, all models had reduced AU-ROCs, but the AU-PRC showed a satisfactory performance (0.80–0.86). The results indicated that AU-PRC may be a good biomarker for predicting COPD.

In addition, the 5 clinical features showed better performance than the SNPs. The AU-ROC and AU-PRC values were above 0.82 and 0.92, respectively (Additional file 9: Fig. S2), with the, PPV (precision), sensitivity (recall) and F1 score exceeding 0.80 for all models; the MCC and specificity for DT and XGboost were above 0.70. KNN had the highest sensitivity (recall) (0.94). Both DT and XGboost had the same highest F1 score (0.93) and the same highest NPV (0.82) (Additional file 10: Table S8). Among all models, the XGboost model also obtained the highest AU-ROC (0.93, 95% CI 0.91–0.95) and AU-PRC (0.97, 95% CI 0.92–0.98), similar to the results shown in Fig. 3, as well as the highest accuracy (0.91), PPV (precision) (0.95), MCC (0.77) and specificity (0.86).

When inputting the top 5 ranked features, the AU-ROC and AU-PRC values were above 0.80 and 0.91, respectively (Additional file 11: Fig. S3), with PPV (precision), sensitivity (recall) and F1 score exceeding 0.82, 0.88 and 0.85 in all models, respectively; among these values, the accuracy values of KNN, LR, DT and XGboost were above 0.81. The MCC, specificity and NPV were above 0.71, 0.77 and 0.80 for both the DT and XGboost models, respectively; the MLP model had the lowest MCC rate (0.43), and the SVM had the lowest specificity rate (0.47) (Additional file 12: Table S9). Moreover, the XGboost model still obtained the highest AU-ROC (0.93, 95% CI 0.88–0.98) and AU-PRC (0.97, 95% CI 0.89–0.99) as well as the highest accuracy (0.91), PPV (precision) (0.94), sensitivity (recall) (0.94), F1 score (0.94), MCC (0.78), specificity (0.84) and NPV (0.84). These results indicated that clinical features played more important roles than SNPs in predicting COPD development.

### Validation of the six models in the test set

**Fig. 5:**
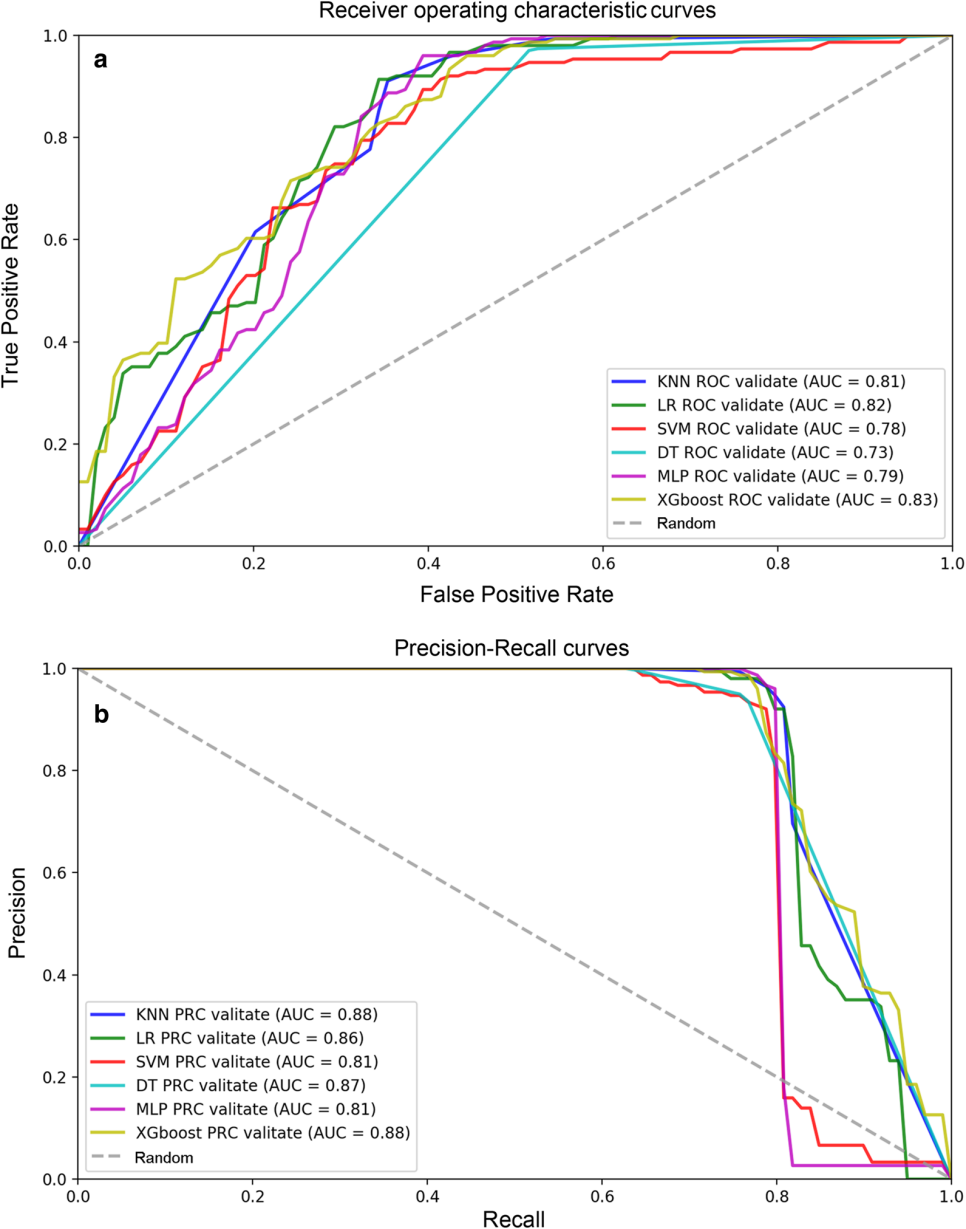
Validation of the training set. **a**, **b** The picture shows the AU-ROC and AU-PRC curves of all models in the test set

According to the training results, we validated all models in the test set. The AU-PRC values were above 0.80 for all models. Among the models, the KNN, LR, and XGboost models had excellent overall predictive power, the AU-ROC values were above 0.80 (Fig. 5), and the accuracy, PPV (precision), sensitivity (recall), F1 score and NPV were above 0.79, 0.78, 0.90, 0.84, and 0.80, respectively. The DT obtained the lowest AU-ROC value (0.73); the MLP model obtained the highest sensitivity (recall) (1.00) and NPV (1.00) but had the lowest specificity rate (0.38) (Table 2).

**Table 2:** The efficacy of KNN, LR, SVM, DT, MLP and XGboost in the test set

Metrics	KNN	LR	SVM	DT	MLP	XGboost
AU-ROC	0.81	0.82	0.78	0.73	0.79	0.83
AU-PRC	0.88	0.86	0.81	0.87	0.81	0.88
accuracy	0.81	0.81	0.78	0.78	0.77	0.80
precision	0.82	0.80	0.77	0.76	0.73	0.79
recall	0.91	0.93	0.93	0.95	1.00	0.93
F1 score	0.86	0.86	0.84	0.85	0.85	0.85
MCC	0.59	0.58	0.52	0.53	0.53	0.56
SPC	0.65	0.60	0.53	0.51	0.38	0.57
NPV	0.81	0.84	0.82	0.85	1.00	0.84

*AU-ROC* area under the receiver operating characteristic curve, *AU-PRC* area under the precision-recall curve, *MCC* Matthews correlation coefficient, *SPC* specificity, *NPV* negative prognostic value, *KNN* k-nearest neighbors classifier, *LR* logistic regression, *SVM* support vector machine, *DT* decision tree, *MLP* multilayer perceptron

In addition, we validated only 9 SNPs, 5 clinical features and the top 5 ranked features in six models. The results indicated that with the 9 SNPs, all models performed poorly, as in the training set (Additional file 13: Table S10), while the recall and F1 score were above 0.89 and 0.73, respectively, for four models (KNN, LR, SVM and MLP). However, the AU-PRC values (0.63–0.81) were higher than the AU-ROC values (0.47–0.50) (Additional file 14: Fig. S4). When only considering 5 clinical features, the AU-PRC values of all models were above 0.79; three models, KNN, LR, and XGboost, had AU-ROC values above 0.81 (Additional file 15: Fig. S5), and the accuracy, PPV (precision), sensitivity (recall), F1 score and NPV were above 0.79, 0.78, 0.92, 0.85 and 0.82, respectively (Additional file 16: Table S11). The SVM had the lowest MCC (0.27) and specificity (0.33) and DT had the lowest AU-ROC (0.73). The MLP obtained the highest sensitivity (recall) (0.99) and NPV (0.98). When inputting the top 5 ranked features, all models had AU-PRC values above 0.80; among these models, KNN, LR, MLP and XGboost all had AU-PRC values above 0.77 (Additional file 17: Fig. S6) as well as an accuracy, PPV (precision), sensitivity (recall), F1 score and NPV above 0.78, 0.77, 0.88, 0.83 and 0.76, respectively. The SVM had the lowest MCC (0.26) and specificity (0.33). DT had the highest recall (0.97) and NPV (0.90), but the lowest AU-ROC (0.73), KNN, LR and XGboost had specificity rates above 0.60 (Additional file 18: Table S12).

## Discussion

COPD is an irreversible and progressive disease, so there is an urgent need to diagnose COPD in the early stage. COPD development is affected by various factors, including genetic susceptibility, air quality, smoking status, age and BMI [12, 13]. However, the combination of genetic polymorphisms and the above factors has not yet been reported to predict early-stage COPD in the Chinese population.

With the development of artificial intelligence, such as machine learning, deep learning, and neural networks, these methods have been successfully used for disease diagnosis and prediction [77–79]. In the present study, we used machine learning to establish risk models (LR, KNN, SVM, MLP, DT and XGboost models) that combined various factors to predict COPD.

In the present study, we employed a dataset with 441 patients with COPD and 192 healthy controls, which satisfied the power analysis. A total of 101 SNPs were identified, and 9 SNPs were significantly associated with COPD development based on PLINK software. Our results showed that 6 SNPs (rs1007052, rs2910164, rs473892, rs161976, rs159497, and rs9296092) were risk factors for COPD, while 3 SNPs (rs8192288, rs20541, and rs12922394) were protective factors for COPD development. These results were roughly shown by previous studies. However, there is controversy regarding the role of individual SNPs in COPD. For example, a GWAS found no significant relationship between rs10007052 and COPD in Europeans [80]. However, rs10007052 was a risk factor for COPD in China [27]. In this study, we also found that rs10007052 was a risk locus for COPD, which provided more evidence for its role in COPD among the Chinese population. The inconsistent results from these studies may result from racial and ethnic differences. A meta-analysis that included 11 studies with 3077 participants (1896 cases and 1181 controls) indicated that the A allele of rs20541 was associated with an elevated risk of COPD in Caucasians but not in Asians [81]. However, in the present study, we found that the A allele of rs20541 (OR = 0.669, *P *< 0.018) was a protective factor for COPD in the Chinese population. More data are needed to validate this finding in the Chinese population.

Apart from SNPs, we also considered 5 clinical features that may be associated with COPD development, such as smoking history, ambient air quality, BMI, age and sex. In the training set, 6 models (LR, KNN, SVM, MLP, DT and XGboost models) were established to predict COPD risk and included 9 SNPs and 5 clinical features. We evaluated the predictive performance of these 6 models for COPD and found that the XGboost model presented the best AU-ROC and AU-PRC values in both the training and test sets in all features. The XGboost algorithm is a highly effective and widely used machine learning method that can build complex models and make accurate decisions when given adequate data [65]. We used the XGboost model to predict feature importance, and the results indicated that the AQCI was the most important factor, while SNPs were less important. This finding was consistent with our knowledge that although COPD development is affected by genetic susceptibility, ambient air pollution and physiological factors may contribute more to the process. In order to verify the importance of clinical features or SNPs for predicting COPD in all models, when only the 9 SNPs were used in all models, we found that the AU-ROC values were very low, but the AU-PRC values were above 0.79. AU-PRC has been reported to provide better agreement with the PPVs of biomarkers and should be preferred over the AU-ROC for evaluating uncommon or rare disease biomarkers [76]. Using unbalanced data, we found that the AU-PRC was a better metric than the AU-ROC, and the fluctuations were also relatively stable. When the models considered only 5 clinical features or the top 5 ranked features combined 3 clinical features and 2 SNPs, both AU-ROC and AU-PRC performed well, similar to all features combining both 5 clinical features and 9 SNPs in the models in the training set. However, in the test set, when inputting the top 5 ranked features, both the AU-ROC and AU-PRC values of the KNN, LR and XGboost models were slightly lower than those of the models that considered only 5 clinical features or combined all features. In addition, when only considering clinical features in the validation set, the accuracy, precision, MCC, specificity and NPV were 0.68, 0.69, 0.27, 0.33 and 0.64 for the SVM model, respectively (Additional file 16: Table S11). When inputting the top 5 ranked features, the accuracy, precision, MCC, specificity and NPV were 0.68, 0.69, 0.26, 0.33 and 0.63 for the SVM model, respectively (Additional file 18: Table S12). When all features, the 5 clinical features and 9 SNPs, were inputted, the accuracy, precision, MMC, specificity and NPV were 0.78, 0.77, 0.52, 0.53 and 0.82 for the SVM model, respectively (Table 2). These results indicated that clinical features played more important role than SNPs in predicting COPD development, while combined all features make various parameters more stable in the models.

There were some limitations in this study. First, the sample size used was relatively small, and the total sample of patients with COPD and healthy controls was unbalanced. Second, the samples from the seven centers were severely unbalanced; in the training set, only control subjects and no COPD patients were collected from the Shanghai area; no control subjects were recruited from Jincheng, and only one COPD patient came from Datong. In the validation set, control subjects and COPD patients from only 5 centers, excluding Jincheng and Shijiazhuang, were enrolled in the test set. Third, we only obtained 9 SNPs for the prediction models, and these SNPs performed worse than the clinical features; there may be other genetic susceptibility SNPs to be discovered. Fourth, we only collected five clinical features for the prediction models, while other risk factors were not collected, such as occupational exposure, childhood chronic cough, parental history of respiratory diseases, and low education in the Chinese population. More work is required before these models can be applied in the clinic for COPD prediction, and these findings should be validated in a larger cohort.

## Conclusions

In conclusion, we identified 9 genetically susceptible loci for COPD and constructed COPD prediction models that comprised SNPs and clinical factors, including ambient air pollution. The KNN, LR and XGboost models showed excellent overall predictive power. We also identified that clinical features were more important than SNPs in predicting COPD development. Our study also revealed that these machine learning tools showed good performance for COPD risk prediction and could potentially be beneficial for the early diagnosis and treatment of patients with COPD in the Chinese population in the near feature.

## Supplementary information

**Additional file 1: Table S1.** The Chinese COPD studies with basic information in international journals.

**Additional file 2: Table S2.** Demographics of COPD patients and control subjectsin the training set.

**Additional file 3: Table S3.** Demographics of COPD patients and control subjectsin the test set.

**Additional file 4: Table S4.** The sequence of 101 SNPs and their primers in multiplex PCR.

**Additional file 5: Table S5.** The parameters selection in the predictive models.

**Additional file 6: Table S6.** Allele frequencies in COPD and control subjects for SNPs.

**Additional file 7: Fig. S1.** Evaluation of the predictive models with only the 9 SNPs as inputs. a, b The picture shows the AU-ROC and AU-PRC curves of the 6 models in the training set. Mean AUC values and 95% CIs of different prediction models are shown in the box.

**Additional file 8: Table S7.** The efficacy of KNN, LR, SVM, DT, MLP and XGboost in the training set of 9 SNPs features.

**Additional file 9: Fig. S2.** Evaluation of the predictive models with only the 5 clinical features as inputs. a, b The picture shows the AU-ROC and AU-PRC curves of the 6 models in the training set. Mean AUC values and 95% CIs of different prediction models are shown in the box.

**Additional file 10: Table S8.** The efficacy of KNN, LR, SVM, DT, MLP and XGboost in the training set of 5 Clinical features.

**Additional file 11: Fig. S3.** Evaluation of the predictive models with the top 5 ranked features as inputs. a, b The picture shows the AU-ROC and AU-PRC curves of the 6 models in the training set. Mean AUC values and 95% CIs of different prediction models are shown in the box.

**Additional file 12: Table S9.** The efficacy of KNN, LR, SVM, DT, MLP and XGboost in the training set of top 5 ranked features.

**Additional file 13: Table S10.** The efficacy of KNN, LR, SVM, DT, MLP and XGboost in the test set of 9 SNPs features.

**Additional file 14: Fig. S4.** Validation of the models in the training set with only the 9 SNPs as inputs. a, b The picture shows the AU-ROC and AU-PRC curves of all models in the test set.

**Additional file 15: Fig. S5.** Validation of the models in the training set with only the 5 clinical features as inputs. a, b The picture shows the AU-ROC and AU-PRC curves of all models in the test set.

**Additional file 16: Table S11.** The efficacy of KNN, LR, SVM, DT, MLP and XGboost in the test set of 5 clinical features.

**Additional file 17: Fig. S6.** Validation of the models in the training set with the top 5 ranked features as inputs. a, b The picture shows the AU-ROC and AU-PRC curves of all models in the test set.

**Additional file 18: Table S12.** The efficacy of KNN, LR, SVM, DT, MLP and XGboost in the test set of top 5 ranked features.

## Data Availability

Data sharing is applicable to this article.

## Abbreviations

COPD: Chronic obstructive pulmonary disease
SNP: Single-nucleotide polymorphism
AQCI: Air Quality Composite Index
FEV1: Forced expiratory volume in one second
FVC: Forced vital capacity
BMI: Body mass index
GWAS: Genome-wide association studies
OR: Odds ratio
LR: Logistic regression
MLP: Multilayer perceptron
DT: Decision tree model
SVM: Support vector machine
KNN: k-Nearest neighbors classifier
AUC: Area under the receiver operating characteristic curve
CI: Confidence interval
PRC: Precision-recall curve
MCC: Matthews correlation coefficient
