# Supplementary material for: Comparison and development of machine learning tools for the prediction of chronic obstructive pulmonary disease in the Chinese population
Source: J Transl Med. 2020 Mar 31;18:146. doi: 10.1186/s12967-020-02312-0 (PMC7110698; doi:10.1186/s12967-020-02312-0)
Supplement: Supplementary file 1 — Additional file 1: Table S1. The Chinese COPD studies with basic information in international journals. [file 12967_2020_2312_MOESM1_ESM.docx]

| **Additional file 1: Table S1 The Chinese COPD studies with basic information in international journals** | | | | | | |
| --- | --- | --- | --- | --- | --- | --- |
| **study** | **Publication**  **year** | **Region of study** | **samples** | **gene** | **SNPs** | **Results** |
| Wang et al[[1](#_ENREF_1)] | 2009 | a Han population in northeastern China | 312 COPD patients and 319 controls | *ADAM33* | rs2787094,rs2280089,rs2280090,rs2280091,rs528557,rs3918396,rs612709,rs511898 | Statistically significant differences in the distributions of the rs2280090G, rs2280091G, rs528557C, and rs612709G alleles between patients and controls were observed (P < 0.001, odds ratio (OR) = 2.81, 95%confidence interval (CI) = 2.19-3.61; P < 0.001, OR = 2.60, 95% CI = 2.06-3.30; P = 0.03, OR = 1.31,95% CI = 1.02-1.69; and P < 0.001, OR = 1.93, 95% CI = 1.50-2.50, respectively). |
| Hosgood et al[[2](#_ENREF_2)] | 2009 | Xuanwei, China | 53 COPD patients and 107 controls | *PTEN* | rs701848 | SNP-based analyses found that homozygote variant carriers of PTEN rs701848 (OR_TT_ = 0.12, 95%CI = 0.03 - 0.47) had a significant decreased risk of COPD. |
| Zhong et al[[3](#_ENREF_3)] | 2009 | Han population sampled from Southwest China | 327 COPD patients and 349 controls | *SERPINE2* | rs3795877, rs6747096, and rs3795879 | Three SNPs (rs3795877, rs6747096, and rs3795879) showed complete linkage disequilibrium (r2 = 1), and the minor allele frequencies were 13.0% and 12.9% in case and control cohorts, respectively, with no significant difference observed (P = 0.96). |
| Yang et al[[4](#_ENREF_4)] | 2010 | Chinese population | 219 COPD patients and 148 controls | *Smad3* | rs28683050，rs28669671，rs2289259，rs28719801，rs28627002，rs2278546，rs10152593，rs7166015 and rs3825977 | The rs28683050 polymorphism frequency of the TT genotype in the COPD patients was significantly higher than that in controls (14.2% versus 5.4%, OR= 3.762, 95% CI =1.650–8.581, P=0.002), and the frequency of the T allele in the COPD patients was significantly higher than that in controls (81.1% versus 66.4%, OR=1.774, 95% CI= 1.354–2.324, P= 0.001). |
| Wang et al[[5](#_ENREF_5)] | 2011 | Chinese Han Population | 409 COPD patients and 411 controls | *SERPINE2* | rs840088, rs1438831and rs3795879 | We found that none of the rs840088G/A, rs1438831G/A and rs3795879 G/A polymorphisms were associated with the disease. The p-values were 0.630, 0.208 and 0.398 respectively. |
| XU et al[[6](#_ENREF_6)] | 2012 | Chinese Han population | 279 COPD patients and 367 controls | *FGF7* | rs10519225 and rs7170426 | The haplotype constructed by allele G at rs10519225 and allele A at rs7170426 was associated with a decreased susceptibility to COPD (P=0.012, OR=0.751, FDR q=0.048) |
| Yuan et al[[7](#_ENREF_7)] | 2012 | Chinese Han population | 279 COPD patients and 367 controls | *ADIPOQ* | rs710445, rs16861205, rs822396, rs7627128, rs1501299, rs3821799 and rs1063537 | The alleles or genotypes of rs1501299 distributed significantly differently in COPD patients and controls (allele: P = 0.002, OR = 1.43 and 95%CI = 1.14–1.79; genotype: P = 0.008) |
| Yang et al[[8](#_ENREF_8)] | 2012 | Southern and eastern Chinese populations. | 1511 COPD patients and 1677 controls | *CHRNA3* | rs6495309 and rs1051730 | the rs6495309CC and rs6495309CT/CC variant genotypes were associated with increased risks of COPD (OR = 1.32, 95% C.I. = 1.14–1.54) |
| Guo et al[[9](#_ENREF_9)] | 2012 | Chinese Han population | 331 COPD patients and 213 controls | *46 genes* | 97 SNPs | Significant differences between patients and healthy controls were observed in the allele frequencies of seven SNPs: rs1205 C(p<0.0223 ,OR=1.34,95%CI= 1.04-1.71; rs2353397 C (P<6.8×10^-20^ ,OR=3.29,95%CI= 2.54-4.28), rs20541 T(p<0.0206,OR= 1.37,95%CI= 1.05-1.79), rs2070600 G(p<0.0043,OR= 1.52,95%CI= 1.14-2.03), rs10947233 G(p<0.0063 OR=1.49,95%CI= 1.12-1.98) , rs1800629 G(p<0.0050,OR= 1.94,95%CI= 1.21-3.10), and rs2241712 A(p<0.0460,OR= 1.28 ,95%CI=1.00-1.64). |
| Wang et al[[10](#_ENREF_10)] | 2012 | Chinese population | 298 COPD patients and 346 controls | *IL-12A and IL-12B* | IL-12A (rs2243115) and IL-12B (rs3212227) | the frequencies of GT and GT + GG of IL-12A rs2243115 were significantly different from TT in the COPD group and the control group (GT vs. TT: odds ratio [OR] = 2.35, 95% confidence interval [CI] = 1.55–3.57, p < 0.001; GT + GG vs. TT: OR = 2.46, 95% CI = 1.63–3.71, p < 0.001) |
| Wang et al[[11](#_ENREF_11)] | 2013 | male smokers of Chinese Han people. | 112 COPD patients and 105 controls | *interleukin (IL)-18 gene promoter* | rs1946518 and rs187238 | Rs1946518 C allele was significantly increased in patients with COPD (p=0.04, OR= 1.48, 95% CI = 1.01–2.15). |
| Deng et al[[12](#_ENREF_12)] | 2013 | Chinese population | 148 COPD patients and 150 controls | *CTLA-4* | rs231775, rs3087243, rs231725 and rs5742909 | none of these four SNPs (rs231775, rs3087243, rs231725, rs5742909) in *CTLA-4* gene were associated with the disease |
| Li et al[[13](#_ENREF_13)] | 2014 | Chinese population | 216 COPD patients and 239 controls | *RAGE* | - 374T/A, - 429T/C, and G82S) | The frequencies of the GS genotype and the S allele in the G82S mutation were significantly higher in COPD patients than in controls (odds ratios [OR] = 1.70, 95% confidence interval [CI]: 1.15–2.50, p = 0.0098 and OR = 1.42, 95% CI: 1.06–1.91, p = 0.023, respectively). Further stratification analysis by smoking status revealed that the presence of the GS genotype conferred a higher risk of developing COPD in current smokers (p = 0.044). |
| Wang et al[[14](#_ENREF_14)] | 2014 | Chinese Han population | 680 COPD patients and 687 controls | *XRCC5* | rs3821104, rs12470053, rs207936, rs3770498, rs6704622,rs3770492, rs4674066, rs7573191, and rs207906 | rs207936 was associated with COPD with an adjusted P value of 0.038, |
| Liu et al[[15](#_ENREF_15)] | 2015 | Chinese Han population | 286 COPD patients and 326 controls | *ABHD2* | rs293379,rs293377,rs16942690,rs293381, rs12442260, rs729707 | The rs12442260 CT/CC genotype was associated with COPD (P < 0.001) under a dominant model. |
| Ding et al[[16](#_ENREF_16)] | 2015 | a Chinese population from Hainan province | 200 COPD patients and 401 controls | *10 genes* | rs1124480,rs7671167,rs10007052,rs1828591,rs13118928,rs13141641,rs13181561,rs3851050,rs954820,rs7937,rs3733829,rs8102683,rs8102683,rs7260329 and rs2823743 | the haplotype analysis revealed that the “CT” haplotype composed of the mutant allele (C) of rs7937, rs3733829 in the EGLN2 gene, was associated with increased COPD risk (P=0.029, OR =1.55; 95% CI= 1.05–2.31) |
| Ding et al[[17](#_ENREF_17)] | 2015 | a Chinese population from Hainan province | 200 COPD patients and 401 controls | *IREB2,HIF1A,CHRNA5* | rs2301104,rs7143164,rs10129270,rs8005745,rs4899056,rs966824,rs10873142,rs2301112,rs2301113,rs4902080,rs13180 and rs66728 | In the genetic model analysis, we found that the genotype T/T of rs13180 of *IREB2decreased the COPD risk by 0.52-fold (P=0.025)* |
| Ding et al[[18](#_ENREF_18)] | 2015 | Chinese Li minority population | 234 COPD patients and 240 controls | *FAM13A, MIR2054, SETD7, RNF150, HHIP and VEGFA* | rs7671167,rs950063,rs17050782,rs10007052,rs1828591,rs13118928,rs13141641,rs25648,rs833068,rs833070,rs3024994,rs3024997,rs3025000,rs3025030,rs3025033 and rs10434 | In genetic models, we found the minor allele of rs7671167 (P=0.028 by dominant model) and rs17050782 (P=0.008 by recessive model) was associated with the increased risk of COPD disease. Likewise, an increased risk of developing COPD was associated with the “GGCGC” haplotype of VEGFA (OR =1.48, 95% CI =1.02–2.12, P=0.037). |
| Yuan et al[[19](#_ENREF_19)] | 2016 | Chinese population | 279 COPD patients and 367 controls | *CDH13* | rs4783244,rs12922394,rs11646011,rs11640875,rs1870843,rs4783266,rs11640522,rs11646849,rs11860282 and rs2549151 | The minor allele T was associated with decreased risk of COPD in the recessive model at rs4783244 (OR=0.42, P=0.023) and in the dominant model at rs12922394 (OR=0.70, P=0.022) |
| Wang et al[[20](#_ENREF_20)] | 2016 | Southern Chinese Population | 594 COPD patients and 600 controls | *BMPR2* | rs6435156 and rs1048829 | Both of them were found associated with significantly increased COPD risk (adjusted odds ratio [OR] = 1.58 with 95% confidence interval [CI] = 1.14–2.15, P = 0.0056 for rs6435156C>T; adjusted OR = 1.47 and 95% CI = 1.10–1.97, P = 0.0092 for rs1048829G>T). |
| Xiong et al[[21](#_ENREF_21)] | 2016 | Chinese Han population | 513 COPD patients and 506 controls | *TRPM8* | rs2362290,rs9789675, rs9789398,rs1003540 and rs1004478 | In the rs9789398 polymorphism, the T/C genotype was associated with an increased risk for COPD (P=0.005); In the rs9789675 polymorphism, the G/A genotype was associated with an increased risk for COPD (P=0.021) |
| Ding et al[[22](#_ENREF_22)] | 2016 | Chinese Han population | 246 COPD patients and 350 controls | *RNF150,VEGFA,ZBTB9-BAK1,SCGB1A1,IREB2,CHRNA5,ATP2C2,TIMM21-CYB5A and EGLN2* | rs10007052,rs25648,rs833068,rs833070,rs3024994,rs3024997,rs3024997,rs3025030,rs3025033,rs10434,rs9296092,rs17157266, rs13180,rs667282,rs8048576,rs9951925,rs7937 and rs7937 | Both rs3025030 and rs3025033 are located on chromosome 6 in VEGF-A. We found one risk allele ‘C’ from rs3025030 and another ‘G’ from rs3025033 (OR =1.40; 95% CI= 1.05-5.96; P = 0.022), (OR= 1.38; 95% CI =1.03–1.84; P = 0.03). We also found another risk allele ‘A’ of rs9296092 in gene region ZBTB9-BAK1 by the allele model (OR= 2.63; 95% CI= 1.27–5.45; P = 0.0078). |
| Ding et al[[23](#_ENREF_23)] | 2017 | Chinese Han population | 279 COPD patients and 290 controls | *RTEL1* | rs6089953,rs6010620 ,rs6010621,rs4809324 and rs2297441 | In the genotype model analysis, we determined that rs4809324 polymorphism had a decreased effect on the risk of COPD (CC versus TT: OR =0.28; 95% CI =0.10–0.82; P=0.02). |
| Deng et al[[24](#_ENREF_24)] | 2017 | Chinese population | 120 COPD patients and 481 controls | *SERPINA1* | rs1243160, rs2854254, and rs8004738 | SNP rs8004738 genotype was associated with a significantly higher risk for COPD (OR =1.835, 95% CI=1.002–3.360), whereas SNPs rs1243160 and rs2854254 did not exhibit such an association. |
| Li et al[[25](#_ENREF_25)] | 2018 | southwest Chinese population | 200 COPD patients and 222 controls | *ADRB2* | rs12654778 | One single nucleotide polymorphism (rs12654778), located upstream of *ADRB2*, a significantly reduced expression of ADRB2 in COPD patients was observed, compared with normal controls (p=0.017). |
| Zhang et al[[26](#_ENREF_26)] | 2018 | Ningxia Hui Autonomous Region | 491 COPD patients and 611 controls | *FAM13A and IREB2* | rs17014601,rs16996144,rs1870339,rs2009746, rs16969858,rs2656065,rs3743079 | Rs17014601 in FAM13A was significantly associated with COPD in the additive (OR=1.36, 95% CI: 1.11–1.67, P=0.003), heterozygote (OR=1.76, 95% CI: 1.33–2.32, P=0.0001), and dominant (OR=1.67, 95% CI: 1.28–2.18, P=0.0001) models. |
| Wang et al[[27](#_ENREF_27)] | 2018 | Chinese Han population | 235 COPD patients and 548 controls | *HIF1A* | rs10873142 | In the genetic model analysis, we found that the TT genotype (TT compared with CC: OR: 1.63; 95% CI=1.02–2.60; P=0.042) and T allele (T compared with C: OR: 1.29; 95%CI, 1.02–1.60; P=0.032) showed significant correlation with the risk of COPD. |
| Ding et al[[28](#_ENREF_28)] | 2019 | a Chinese population from Hainan province | 318 COPD patients and 508 controls | *CYP2B6* | rs12979270,rs4803420,rs2099361,rs1038376 and rs4803418 | In allele model, we observed that rs4803420 G and rs1038376 A were related to COPD risk. And rs4803420 G/T and G/T-T/T were related to a decreased COPD risk compared to GG genotype in the co-dominant and dominant models, respectively. When comparing with the AA genotype, rs1038376 A/T and A/T-T/T were associated with an increased COPD risk in the co-dominant and dominant models, respectively. |
| Ding et al[[29](#_ENREF_29)] | 2019 | Chinese Li population | 279 COPD patients and 290 controls | *TERT* | rs2075786,rs10069690,rs2242652,rs2853677, and rs2853676 | In the genetic model analysis, the “C/T‐T/T” genotype of rs10069690 in TERT was associated with an increased COPD risk in the dominant model (p = 0.046); the rs2853677 in TERT was significantly associated with increased COPD risk based on the codominant model (“A/G” genotype, p = 0.033), dominant model (A/G‐G/G genotype, p = 0.0091), and log additive model (p = 0.023). The rs2853676 in TERT could increase the risk of COPD in the dominant model (“C/T‐T/T” genotype, p = 0.026) and in the Log-additive model (p = 0.022). |
| Li et al[[30](#_ENREF_30)] | 2019 | Chinese Han population | 152 COPD patients and 1727 controls | *TLR4* | rs10759932, rs2737190, rs7873784, rs11536889, and rs10983755 | Compared with rs10759932-TT, individual scurrying TC (OR: 0.42, 95% CI: 0.28–0.64) or CC (OR: 0.24, 95% CI: 0.09–0.63) had a significantly reduced risk of COPD; For rs2737190, heterozygous AG was related to a decreased risk of COPD (OR: 0.32, 95% CI: 0.21–0.49). |

References:

1. Wang X, Li L, Xiao J, Jin C, Huang K, Kang X, Wu X, Lv F: **Association of ADAM33 gene polymorphisms with COPD in a northeastern Chinese population.** *BMC Med Genet* 2009, **10:**132.

2. Hosgood HD, 3rd, Menashe I, He X, Chanock S, Lan Q: **PTEN identified as important risk factor of chronic obstructive pulmonary disease.** *Respir Med* 2009, **103:**1866-1870.

3. Zhong L, Fu WP, Sun C, Dai LM, Zhang YP: **Absence of association between SERPINE2 genetic polymorphisms and chronic obstructive pulmonary disease in Han Chinese: a case-control cohort study.** *BMC Med Genet* 2009, **10:**66.

4. Yang T, Ying B, Song X, Zhang S, Fan H, Xu D, Wang T, Liu D, Wen F: **Single-nucleotide polymorphisms in SMAD3 are associated with chronic obstructive pulmonary disease.** *Exp Biol Med (Maywood)* 2010, **235:**599-605.

5. Wang A, Yin Y, Chen P, Liu Q, Yu Q, Xiao W: **The association of SERPINE2 gene with COPD in a Chinese Han population.** *Yonsei Med J* 2011, **52:**953-960.

6. Xu SC, Kuang JY, Liu J, Ma CL, Feng YL, Su ZG: **Association between fibroblast growth factor 7 and the risk of chronic obstructive pulmonary disease.** *Acta Pharmacol Sin* 2012, **33:**998-1003.

7. Yuan Y, Jiang H, Kuang J, Hou X, Feng Y, Su Z: **Genetic variations in ADIPOQ gene are associated with chronic obstructive pulmonary disease.** *PLoS One* 2012, **7:**e50848.

8. Yang L, Qiu F, Lu X, Huang D, Ma G, Guo Y, Hu M, Zhou Y, Pan M, Tan Y, et al: **Functional polymorphisms of CHRNA3 predict risks of chronic obstructive pulmonary disease and lung cancer in Chinese.** *PLoS One* 2012, **7:**e46071.

9. Guo Y, Gong Y, Pan C, Qian Y, Shi G, Cheng Q, Li Q, Ren L, Weng Q, Chen Y, et al: **Association of genetic polymorphisms with chronic obstructive pulmonary disease in the Chinese Han population: a case-control study.** *BMC Med Genomics* 2012, **5:**64.

10. Wang EY, Liang WB, Zhang L: **Association between single-nucleotide polymorphisms in interleukin-12A and risk of chronic obstructive pulmonary disease.** *DNA Cell Biol* 2012, **31:**1475-1479.

11. Wang J, Liu X, Xie J, Xu Y: **Association of interleukin-18 promoter polymorphisms with chronic obstructive pulmonary disease in male smokers.** *International Journal of Immunogenetics* 2013, **40:**204-208.

12. Deng L, Zhou H, Yang J, Xiao J, Wang B, Wang L, Ou X, Feng Y: **CTLA-4 gene polymorphisms and susceptibility to chronic obstructive pulmonary disease.** *Int J Clin Exp Pathol* 2013, **6:**2548-2553.

13. Li Y, Yang C, Ma G, Gu X, Chen M, Chen Y, Zhao B, Cui L, Li K: **Association of polymorphisms of the receptor for advanced glycation end products gene with COPD in the Chinese population.** *DNA Cell Biol* 2014, **33:**251-258.

14. Wang B, Yang J, Xiao J, Liang B, Zhou HX, Su Z, Xu S, Chen H, Ma C, Deng J, et al: **Association of XRCC5 polymorphisms with COPD and COPD-related phenotypes in the Han Chinese population: a case-control cohort study.** *Genet Mol Res* 2014, **13:**7070-7078.

15. Liu L, Li X, Yuan R, Zhang H, Qiang L, Shen J, Jin S: **Associations of ABHD2 genetic variations with risks for chronic obstructive pulmonary disease in a Chinese Han population.** *PLoS One* 2015, **10:**e0123929.

16. Ding Y, Niu H, Yang H, Sun P, Chen Y, Duan M, Xu D, Xu J, Jin T: **EGLN2 and RNF150 genetic variants are associated with chronic obstructive pulmonary disease risk in the Chinese population.** *Int J Chron Obstruct Pulmon Dis* 2015, **10:**145-151.

17. Ding Y, Yang D, Xun X, Wang Z, Sun P, Xu D, He P, Niu H, Jin T: **Association of genetic polymorphisms with chronic obstructive pulmonary disease in the Hainan population: a case-control study.** *Int J Chron Obstruct Pulmon Dis* 2015, **10:**7-13.

18. Ding Y, Yang D, Zhou L, Xu J, Chen Y, He P, Yao J, Chen J, Niu H, Sun P, Jin T: **Variants in multiple genes polymorphism association analysis of COPD in the Chinese Li population.** *Int J Chron Obstruct Pulmon Dis* 2015, **10:**1455-1463.

19. Yuan YM, Zhang JL, Xu SC, Ye RS, Xu D, Zhang Y, Zhang YJ, Chen YL, Liu YL, Su ZG: **Genetic variants of CDH13 determine the susceptibility to chronic obstructive pulmonary disease in a Chinese population.** *Acta Pharmacol Sin* 2016, **37:**390-397.

20. Wang J, Zhang C, Zhang Z, Zheng Z, Sun D, Yang Q, Hadadi C, Li D, Xu X, Xiong M, et al: **A Functional Variant rs6435156C > T in BMPR2 is Associated With Increased Risk of Chronic Obstructive Pulmonary Disease (COPD) in Southern Chinese Population.** *EBioMedicine* 2016, **5:**167-174.

21. Xiong M, Wang J, Guo M, Zhou Q, Lu W: **TRPM8 genetic variations associated with COPD risk in the Chinese Han population.** *Int J Chron Obstruct Pulmon Dis* 2016, **11:**2563-2571.

22. Ding Y, Niu H, Li Y, He P, Li Q, Ouyang Y, Li M, Hu Z, Zhong Y, Sun P, Jin T: **Polymorphisms in VEGF-A are associated with COPD risk in the Chinese population from Hainan province.** *J Genet* 2016, **95:**151-156.

23. Ding Y, Xu H, Yao J, Xu D, He P, Yi S, Li Q, Liu Y, Wu C, Tian Z: **Association between RTEL1 gene polymorphisms and COPD susceptibility in a Chinese Han population.** *Int J Chron Obstruct Pulmon Dis* 2017, **12:**931-936.

24. Deng X, Yuan CH, Chang: **Interactions between single nucleotide polymorphism of SERPINA1 gene and smoking in association with COPD: a case-control study.** *Int J Chron Obstruct Pulmon Dis* 2017, **12:**259-265.

25. Li JX, Fu WP, Zhang J, Zhang XH, Sun C, Dai LM, Zhong L, Yu L, Zhang YP: **A functional SNP upstream of the ADRB2 gene is associated with COPD.** *Int J Chron Obstruct Pulmon Dis* 2018, **13:**917-925.

26. Zhang Y, Qiu J, Zhang P, Zhang J, Jiang M, Ma Z: **Genetic variants in FAM13A and IREB2 are associated with the susceptibility to COPD in a Chinese rural population: a case-control study.** *Int J Chron Obstruct Pulmon Dis* 2018, **13:**1735-1745.

27. Wang L, Tang Y, Chen Y: **HIF1A gene rs10873142 polymorphism is associated with risk of chronic obstructive pulmonary disease in a Chinese Han population: a case-control study.** *Biosci Rep* 2018, **38**.

28. Ding Y, Li Q, Feng Q, Xu D, Wu C, Zhao J, Zhou X, Yang Y, Niu H, He P, Xing L: **CYP2B6 genetic polymorphisms influence chronic obstructive pulmonary disease susceptibility in the Hainan population.** *Int J Chron Obstruct Pulmon Dis* 2019, **14:**2103-2115.

29. Ding Y, Li Q, Wu C, Wang W, Zhao J, Feng Q, Zhou X, Xie Y, Lin M, He P, Xie P: **TERT gene polymorphisms are associated with chronic obstructive pulmonary disease risk in the Chinese Li population.** *Mol Genet Genomic Med* 2019, **7:**e773.

30. Li Z, Mao X, Liu Q, Song H, He B, Shi P, Zhang Q, Li X, Wang J: **Functional variations of the TLR4 gene in association with chronic obstructive pulmonary disease and pulmonary tuberculosis.** *BMC Pulm Med* 2019, **19:**184.
