# Supplementary material for: Comparison and development of machine learning tools for the prediction of chronic obstructive pulmonary disease in the Chinese population
Source: J Transl Med. 2020 Mar 31;18:146. doi: 10.1186/s12967-020-02312-0 (PMC7110698; doi:10.1186/s12967-020-02312-0)
Supplement: Supplementary file 2 — Additional file 2: Table S2. Demographics of COPD patients and control subjectsin the training set. [file 12967_2020_2312_MOESM2_ESM.docx]

**Additional file 2: Table S2** Demographics of COPD patients and control subjects
in the training set

|  | | | | | | | | | | | | | |
| --- | --- | --- | --- | --- | --- | --- | --- | --- | --- | --- | --- | --- | --- |
| **Groups** | **centers** | **ZIP**  **Code** | **AQCI** | **age** | **sex** | M | F | **smokig status** | smoking | never smoking | **BMI** | **FEV1/FVC**  **(%)** | **FEV1**  **(%）** |
| **control** | SH | 201204 | 4.63 | 55.50  (26-81) | 42 | 23 | 19 | **42** | 15 | 27 | 23.19  (19.22-33.30) | 82.58  (75.81-108.00) | 94.89  (79.00-118.00) |
|  | DT | 037000 | 5.31 | 36 | 1 | 1 | 0 | **1** | 0 | 1 | 23.15 | 77.16 | 90 |
|  | CZ | 046011 | 6.88 | 53  (40-60) | 31 | 26 | 5 | **31** | 16 | 15 | 25.70  (18.20-29.50) | 84.10  （61.50-93.40） | 100.00  (57.98-116.00) |
|  | TY | 030008 | 7.76 | 50  (40-67) | 16 | 9 | 7 | **16** | 7 | 9 | 21.97  (20.06-24.88) | 78.14  （72.15-95.50） | 85.83  (81.24-113.20) |
|  | LF | 041000 | 8.6 | 53.50  (27-69) | 8 | 2 | 6 | **8** | 1 | 7 | 22.03  (18.80-28.65) | 80.20  (72.60-90.23) | 92.45  (82.30-106.30) |
|  | SJZ | 050000 | 8.72 | 73  (58-80) | 5 | 4 | 1 | **5** | 4 | 1 | 22.34  (18.30-27.00) | 87.06  （68.10-90.20） | 94.40  (92.13-109.50) |
|  | **total** |  | 7.32  (4.63-8.72) | 53  (26-81) | **103** | 65 | 38 | **103** | 43 | 60 | 23.44  (18.20-33.30) | 82.36  (61.50-108.00) | 94.52  (57.98-118.00) |
| **COPD** | DT | 037000 | 5.31 | 71.50  (26-89) | 86 | 70 | 16 | **86** | 64 | 22 | 23.84  (15.57-43.56) | 61.00  (36.51-70.00) | 55.00  (26.35-86.89) |
|  | CZ | 046011 | 6.88 | 70.00  (49-86) | 23 | 16 | 7 | **23** | 13 | 10 | 21.11  (16.70-28.40) | 50.60  (25.23-71.90) | 41.00  (18.20-88.50) |
|  | TY | 030008 | 7.76 | 66  (51-84) | 50 | 38 | 12 | **50** | 38 | 12 | 22.60  (15.04-34.00) | 59.00  (30.02-78.65) | 48.60  (16.40-94.70) |
|  | JC | 048000 | 7.78 | 68  (53-75) | 15 | 15 | 0 | **15** | 15 | 0 | 22.99  (18.20-24.16) | 42.29  (31.21-67.18) | 31.90  (21.60-65.70) |
|  | LF | 041000 | 8.6 | 70  (46-86) | 53 | 47 | 6 | **53** | 43 | 10 | 21.50  (16.32-30.78) | 57.60  (40.28-83.57) | 68.00  (27.90-79.00) |
|  | SJZ | 050000 | 8.72 | 66  (39-86) | 63 | 47 | 16 | **63** | 43 | 20 | 23.50  (15.60-35.70) | 57.00  (25.23-68.50) | 54.00  (20.02-101.20) |
|  | **total** |  | 7.77  (5.31-8.72) | 68  (26-89) | **290** | 233 | 47 | **290** | 216 | 74 | 23.12  (15.04-43.56) | 57.95  (25.23-83.57) | 56.74  (16.40-101.20) |
| ***P*** |  |  |  | ***<0.0001*** | ***<0.0001*** |  |  | ***<0.0001*** |  |  | **0.08** | ***<0.0001*** | ***<0.0001*** |

*****P<0.0001, nonparametric Mann–Whitney U test or chi-squared test;*

COPD, Chronic obstructive pulmonary disease; AQCI, Air Quality Composite Index; FEV1, forced expiratory volume in one second ; FVC, forced vital capacity; BMI, body mass index; M, male; F, female；SH, Shanghai；DT, Datong；CZ, Changzhi; TY, Taiyuan; LF, Linfen; SJZ, Shijiazhuang.
