# Supplementary material for: Comparison and development of machine learning tools for the prediction of chronic obstructive pulmonary disease in the Chinese population
Source: J Transl Med. 2020 Mar 31;18:146. doi: 10.1186/s12967-020-02312-0 (PMC7110698; doi:10.1186/s12967-020-02312-0)
Supplement: Supplementary file 3 — Additional file 3: Table S3. Demographics of COPD patients and control subjectsin the test set. [file 12967_2020_2312_MOESM3_ESM.docx]

| **Additional file 3: Table S3 Demographics of COPD patients and control subjects in thetest set** | | | | | | | | | | | | | |
| --- | --- | --- | --- | --- | --- | --- | --- | --- | --- | --- | --- | --- | --- |
| **groups** | **centers** | **ZIP**  **Code** | **AQCI** | **age** | **sex** | M | F | **smoking status** | smoking | never smoking | **BMI** | **FEV1/FVC**  **(%)** | **FEV1**  **(%）** |
| **control** | SH | 201204 | 4.63 | 27 (24-44） | **19** | 6 | 13 | **19** | 3 | 16 | 21.48  (17.15-24.74) | 82.60  (75.63-95.65) | 90.20  (80.35-112.36) |
|  | DT | 037000 | 5.31 | 67(24-94) | **49** | 22 | 27 | **49** | 24 | 25 | 23.30  (16.36-31.40) | 83.67  (76.32-100.7) | 92.30  (80.00-112.30) |
|  | TY | 030008 | 7.76 | 29(22-33) | **21** | 5 | 16 | **21** | 1 | 20 | 22.31  (18.43-29.38) | 80.32  (76.35-86.35) | 86.45  (81.35-103.2) |
|  | **total** |  | 5.31  (4.63-7.76) | 46  (21-94) | **89** | 33 | 56 | **89** | 28 | 61 | 22.49  (16.36-31.40) | 83.21  (75.63-100.70) | 91.23  (80.00-112.36) |
| **COPD** | DT | 037000 | 5.31 | 73 (44-98) | **118** | 102 | 16 | **118** | 91 | 27 | 23.02  (14.30-31.39) | 56.67  (29.86-90.20) | 61.10  (22.10-108.70) |
|  | CZ | 046011 | 6.88 | 70 (50-81) | **4** | 3 | 1 | **4** | 2 | 2 | 24.87  (24.22-27.34) | 57.23  (42.26-63.21) | 58.13  (28.95-70.20) |
|  | LF | 041000 | 8.6 | 72 (49-89) | **29** | 25 | 4 | **29** | 20 | 9 | 21.77  (15.82-29.38) | 63.00  (50.46-74.98） | 68.00  (52.00-83.54） |
|  | **total** |  | 6.88  (5.31-8.60) | 73  (44-98) | **151** | 130 | 21 | **151** | 113 | 38 | 23.03  (14.30-31.39) | 60.00  (29.86-90.20) | 63.32  (22.10-108.70) |
| ***p*** |  |  |  | ***<0.0001*** | ***<0.0001*** |  |  | ***<0.0001*** |  |  | ***0.81*** | ***<0.0001*** | ***<0.0001*** |

­­­

****P<0.0001, t-test was used in BMI level; nonparametric Mann–Whitney U test or chi-squared test;*

COPD, Chronic obstructive pulmonary disease; AQCI, Air Quality Composite Index; FEV1, forced expiratory volume in one second; FVC, forced vital capacity; BMI, body mass index; M, male; F, female；SH, Shanghai; DT, Datong; CZ, Changzhi; TY, Taiyuan; LF, Linfen; SJZ, Shijiazhuang.
