# Supplementary material for: Comparison and development of machine learning tools for the prediction of chronic obstructive pulmonary disease in the Chinese population
Source: J Transl Med. 2020 Mar 31;18:146. doi: 10.1186/s12967-020-02312-0 (PMC7110698; doi:10.1186/s12967-020-02312-0)
Supplement: Supplementary file 4 — Additional file 4: Table S4. The sequence of 101 SNPs and their primers in multiplex PCR. [file 12967_2020_2312_MOESM4_ESM.docx]

Additional file 4: Table S4 **The sequence of 101 SNPs and their primers in multiplex PCR**

| SNPs | Chr | Position | gene | Primers (5’-3’) | | |
| --- | --- | --- | --- | --- | --- | --- |
|  |  |  |  | 1st-PCR primer | 2nd-PCR primer | UEP_SEQ |
| rs10007052 | 4 | 141084419 | RNF150 | ACGTTGGATGAAAAGAGGAAATCTGCAGAG | ACGTTGGATGGTTTCCATGGTTGACATAAT | ATTGTATTCCTAGTATTTACCTT |
| rs8192288 | 4 | 24795056 | SOD3 | ACGTTGGATGCCTCACACCCCCATTTTACA | ACGTTGGATGACTTCCTATCTGTGTACGCC | GTGTACGCCTGAAGCAG |
| rs20541 | 5 | 132660272 | IL-13 | ACGTTGGATGTTCAATAGTCAGGTCCTGTC | ACGTTGGATGCCCAGTTTGTAAAGGACCTG | TTTTTCGCGAGGGAC |
| rs12922394 | 16 | 82638722 | CDH13 | ACGTTGGATGTCCCATATTAGTAGGTCCCC | ACGTTGGATGAGAAGATGAGATCCCACCAG | CCCGGATTTTATGAGGCATGAATCCC |
| rs2910164 | 5 | 160485411 | MIR146A | ACGTTGGATGCACGATGACAGAGATATCCC | ACGTTGGATGAAGCCGATGTGTATCCTCAG | TGTCAGTGTCAGACCT |
| rs161976 | 12 | 32215232 | BICD1 | ACGTTGGATGATTCTATCCTGGGTGGCAAG | ACGTTGGATGAAGGGATGAGGTGGAATGAC | CCTTTTCACATGGTTCTTTTTTC |
| rs473892 | 6 | 137629910 | Intergenic | ACGTTGGATGACTTTCTACTCCTGCATGGG | ACGTTGGATGGTGCCAAAAAGGTTGGGAGC | CTCTCCACTGCTATAGAGGGTTC |
| rs159497 | 5 | 58912391 | Intergenic | ACGTTGGATGCTACCCAACTGTTTTTCCAC | ACGTTGGATGTCTGTCTTGTTTCCTTGCCC | TCTATCTTGTAAAGAGCCTTCAT |
| rs9296092 | 6 | 33510719 | Intergenic | ACGTTGGATGGTAAGCAATGAAACAGACAC | ACGTTGGATGGACTATTAAAGCCTAGCTCC | GGGGAACACTGAAAAGCGTGTA |
| rs1690139 | 12 | 75878897 | LOC105369844 | ACGTTGGATGCCTTCCCTAAATTTGTACCC | ACGTTGGATGGTCACATACCTTCCTTAGGC | TAAGGTTTTCTATCTACAAAAGC |
| rs4148382 | 16 | 16144637 | Intergenic | ACGTTGGATGGACATCATTCAGGCATGAGC | ACGTTGGATGAGTGAAAAGCCAGTAAGGTC | GGTCACCATTAGAATAGGTAGTATCA |
| rs3024791 | 2 | 85668581 | SFTPB | ACGTTGGATGTGGGTGTTCCCCTCCCATC | ACGTTGGATGCAGGAAGCTCTCAAGAGCAT | CCCGCTCAAGAGCATTGCTCAA |
| rs4073 | 4 | 73740307 | CXCL8 | ACGTTGGATGTGTTCTAACACCTGCCACTC | ACGTTGGATGCTGAAGCTCCACAATTTGGT | CCCCACAATTTGGTGAATTATCAA |
| rs915895 | 6 | 32222440 | NOTCH4 | ACGTTGGATGTGTCTTCAAAGGGCTTGCAG | ACGTTGGATGAGCAATGGGCTAGGCTGTG | AAGCGGTGGGTGGGGAAGAT |
| rs1542725 | 4 | 144567182 | Intergenic | ACGTTGGATGCCACCTCTTAAGACTAGTGC | ACGTTGGATGTTGGTAAGTGCTGTGGTCTG | GGGGCTGAATGTTTGTGTCCC |
| rs10461985 | 5 | 37874307 | GDNF-AS1 | ACGTTGGATGAGAAGCGAAAACTTCTGAGG | ACGTTGGATGACACACTCTCTCTCTAGACG | CTCTAGACGTAAATATATAAAATCATG |
| rs17050782 | 4 | 139501980 | SETD7 | ACGTTGGATGAGGCGGAGCAAAGTTAGTGA | ACGTTGGATGAAACTGTGATGACAACTGGG | AAAAGATTGAGTTTTGACCCA |
| rs1130866 | 2 | 85666618 | SFTPB | ACGTTGGATGTGTGTGTGTGGCTCCCCAT | ACGTTGGATGTGGTCATCGACTACTTCCAG | ACTACTTCCAGAACCAGA |
| rs1800630 | 6 | 31574699 | TNF | ACGTTGGATGGCTATGGAAGTCGAGTATGG | ACGTTGGATGGTATTCCATACCTGGAGGTC | CCTCGACATGGCCCTGTCTTCGTTAAG |
| rs181206 | 16 | 28502082 | IL27 | ACGTTGGATGGCTGCATCCTCTCCATGTTG | ACGTTGGATGTTCATCTCCACCACGCTTCA | AGCCCTTCCATGCCC |
| rs1801282 | 3 | 12351626 | PPARG | ACGTTGGATGTGTATCAGTGAAGGAATCGC | ACGTTGGATGCAAACCCCTATTCCATGCTG | TGGGAGATTCTCCTATTGAC |
| rs11677877 | 2 | 227266453 | COL4A3 | ACGTTGGATGGACATCGTTTTTCGCAAGGG | ACGTTGGATGTTCCTGGAGACCCTAGATAG | GGGCTAGCCTGGCAGTCCG |
| rs911887 | 10 | 79941767 | SFTPD | ACGTTGGATGGGGACTGTTCCTATCTAGGG | ACGTTGGATGCCACCCAACAACTAACCTTC | TCCATTGCTTGCGCC |
| rs6609533 | 23 | 47585887 | TIMP-1 | ACGTTGGATGCAAGGGAGAATTAGAGGGAC | ACGTTGGATGTTTCCCTGTGTCCAATACCG | CTCCATGTGTCCAATACCGTGTGATC |
| rs2243115 | 3 | 159988493 | IL12A-AS1/ IL12A | ACGTTGGATGTAATTGCAGAGTCAGCCAGC | ACGTTGGATGGGGCATTGCCACACTAAATC | CCGCCACACTAAATCAGACTC |
| rs1051740 | 1 | 225831932 | EPHX1 | ACGTTGGATGCTGGCGTTTTGCAAACATAC | ACGTTGGATGGGCGGAATGAATTTGACTGG | GTGGAGATTCTCAACAGA |
| rs7041 | 4 | 71752617 | GC | ACGTTGGATGTTTTTCAGACTGGCAGAGCG | ACGTTGGATGGCTTGTTAACCAGCTTTGCC | GGGTGGTTCCGTGGGTGTGGC |
| rs231775 | 2 | 203867991 | CTLA4 | ACGTTGGATGAAAAACAGGAGAGTGCAGGG | ACGTTGGATGTTGGATTTCAGCGGCACAAG | CCAGCTGAACCTGGCT |
| rs8034191 | 15 | 78513681 | HYKK | ACGTTGGATGAGTGGTTAGAGCCCAATGTG | ACGTTGGATGCCACAAGTCCCCTTAGTTAC | CCCCCTCTGTCAGGGCCTTTCT |
| rs3733829 | 19 | 40804666 | RAB4B-EGLN2 | ACGTTGGATGCATCCACGTGCATCATCAGT | ACGTTGGATGGGAGGCTTAGAAACAGTGAG | AGTGAGTTACTGACTAGAGA |
| rs2234922 | 1 | 225838705 | EPHX1 | ACGTTGGATGCACTTCATCCACGTGAAGCC | ACGTTGGATGAAAACTCGTAGAAAGAGCCG | GGAAGGGCTTCGGGGTA |
| rs2070600 | 6 | 32183666 | AGER | ACGTTGGATGTTGCCTGGCACCGGAAAATC | ACGTTGGATGACAGTGTGGCTCGTGTCCTT | CGTGTCCTTCCCAAC |
| rs35621 | 16 | 16074751 | ABCC1 | ACGTTGGATGGGAAGGTGCTGGTTAACCTA | ACGTTGGATGTCCCAGTTTCCTCATTCCAC | TTTTCTCCTCATTCCACGTCCACT |
| rs25882 | 5 | 132075767 | CSF2 | ACGTTGGATGAGAAAGTCCTTCAGGTTCTC | ACGTTGGATGTTAAAGGAAACTTCCTGTGC | TCTGCCTGTGCAACCCAGA |
| rs17111652 | 1 | 55124792 | USP24 | ACGTTGGATGTTTCTCAAGCTGCCATCCTG | ACGTTGGATGCCACAGCATGAAGATCAATC | AGCTGTTTTACAGATAAAAAAGAGA |
| rs2276109 | 11 | 102875061 | MMP12 | ACGTTGGATGATAGCCCTTAGTCCGGGTTC | ACGTTGGATGTTGAGATAGATCAAGGGATG | AAAGCTAGATCAAGGGATGATATCAACT |
| rs10473352 | 5 | 44308150 | FGF10 | ACGTTGGATGGCTCATGGACTGTGCTTATG | ACGTTGGATGAATAGAAAGCCACCGGACTC | CCCCCCACCGGACTCTACTTTAA |
| rs1011814 | 5 | 44335718 | FGF10 | ACGTTGGATGCTTTCCCAATCTGTATAGTC | ACGTTGGATGCAAGCCATGATTAGAGTTGC | CTTGGCCTGTCTAGCTTTAATATAC |
| rs4863687 | 4 | 139757127 | MAML3 | ACGTTGGATGGGTAAAACGCCCTGATTCTC | ACGTTGGATGCATCTTGCTAGCCTGATGTC | ACTCTTCATCTTCTGCC |
| rs2604894 | 19 | 40786499 | MIA-RAB4B | ACGTTGGATGAGCTGAGCTCTGAAGGAAGA | ACGTTGGATGTTTCCCTGCCACTAATGTCC | GGCATGTCCGCTTCCCGA |
| rs9863587 | 3 | 197854276 | LRCH3 | ACGTTGGATGGATGATTGTGAATGTGGTCC | ACGTTGGATGGCACACGTGTGACGTATTTC | ATTAAGACATACTTTTGAAGACG |
| rs734556 | 2 | 223696612 | Intergenic | ACGTTGGATGACACATAGACTCAGATGTCC | ACGTTGGATGTTTTGTGGATGAGTGTGGGC | GACTCCTGCTCTCTCCCCTTA |
| rs528557 | 20 | 3671095 | ADAM33 | ACGTTGGATGAGTCGGTAGCAACACCAGG | ACGTTGGATGAGACCATGACACCTTCCTGC | CGCCTCTGCTCCCAGG |
| rs2020936 | 17 | 30223796 | SLC6A4 | ACGTTGGATGCCAGGCTCAAGAATGCAAAC | ACGTTGGATGAGCATAAATGGTGAGCAGGG | CCGGGTGAGGTTATGGAGA |
| rs1042713 | 5 | 148826877 | ADRB2 | ACGTTGGATGATGAGAGACATGACGATGCC | ACGTTGGATGGAACGGCAGCGCCTTCTTG | TTCTTGCTGGCACCCAAT |
| rs6577641 | 3 | 18356357 | SATB1 | ACGTTGGATGGTGCAAATGTCTTGCTACCG | ACGTTGGATGATCCCTAACCAAAACCAGTC | ACAGTCAAAAGAATGTTATTTATTTC |
| rs11779254 | 8 | 4391552 | CSMD1 | ACGTTGGATGTTGGTCTGGAGTCAAAAGCG | ACGTTGGATGCACAACCACTGCATCAACCA | AGAGTCAACCAGCTCAGAGTA |
| rs660652 | 15 | 78595490 | CHRNA3 | ACGTTGGATGAACCAGGGGCAATTTTGCTC | ACGTTGGATGATGCCAATGACATACCTTGC | AACAAAGAATGTCTCCATTGTTAAATGT |
| rs16878037 | 5 | 60413925 | PDE4D | ACGTTGGATGAGGGCATCGGTTCTGTTTTC | ACGTTGGATGCTAACCCAAAGAACACTTCC | GATTTATCAGAGTTCAGGTTCA |
| rs3995090 | 5 | 148466252 | HTR4 | ACGTTGGATGCCAAGTAAGGAAACTCAGCC | ACGTTGGATGGACTATATGGAGGAAGTGGC | TGCAACATAGACATATCATGGAGAT |
| rs584367 | 1 | 20115561 | PLA2G2D | ACGTTGGATGACTCACAGCAGTGGATGTTC | ACGTTGGATGTGCTATGACCACCTGAAGAC | AACAAAGACCCAGGGGTGC |
| rs17576 | 20 | 46011586 | MMP9 | ACGTTGGATGTATAATGTGCTGTCTCCGCC | ACGTTGGATGAACTGGCAGGGTTTCCCATC | AAACCTCAGCATTGCCGTCC |
| rs10859974 | 12 | 95895082 | CCDC38 | ACGTTGGATGTCCTCCTCAGGGAGTATATG | ACGTTGGATGTAGTGCTTGCTGGATTTGCC | GCCAATGTTTTGGAGACA |
| rs7181486 | 15 | 78449276 | IREB2 | ACGTTGGATGCCGTATCCAGTTCTATAAGC | ACGTTGGATGCCACCACTTAGTAGGTAACG | CCCCTGGACCTTGGCCAAGTGT |
| rs13278529 | 8 | 40475802 | Intergenic | ACGTTGGATGCATTGTCTTGCAGCTCTGTG | ACGTTGGATGTGTCCTCCCTTGATAGAGTG | CCTTGATAGAGTGACTAAAAGA |
| rs1903003 | 4 | 88965146 | FAM13A | ACGTTGGATGGGCCTAATGCTCATTGTCTG | ACGTTGGATGTGAACAGGCAAGACTAAGGC | AATGGTACAATTTGAGCAG |
| rs3773445 | 3 | 25571074 | RARB | ACGTTGGATGCAATAATGGTGACCAGGAGC | ACGTTGGATGAGCCACCTAGGTTTGTGCTG | TGGCCCCAGATTCCA |
| rs3088308 | 10 | 79938112 | SFTPD | ACGTTGGATGAACCATTTACGGAGGCACAG | ACGTTGGATGTTGCAAGGCGGCATTCTCAG | AGCGGCAGAGCGTGGAG |
| rs10519225 | 15 | 49428581 | FAM227B | ACGTTGGATGCAGAGGCTATCATTCTGCAC | ACGTTGGATGTGCTGCTTTCTCTCACTCGG | CACTCGGCACCTCAATA |
| rs2202507 | 4 | 144336529 | LOC105377462 | ACGTTGGATGTAACTCATGCAGCTTCACAG | ACGTTGGATGAGTTTTCCCACTCTGAGGTC | GACGACACTAGTTTTTAAAGTTTT |
| rs701848 | 10 | 87966988 | PTEN | ACGTTGGATGGTATGCAGTCTGGGCATATC | ACGTTGGATGTTTCTTCATAGTGCTCCCCC | CAGTTGGGACTAGGGC |
| rs3025033 | 6 | 43783338 | VEGFA | ACGTTGGATGATCCCCTGAGCACACACAAG | ACGTTGGATGCTGTGTTAGGGAAGTCCTTG | CTCCCCTCCCCCAGC |
| rs6435156 | 2 | 202560752 | BMPR2 | ACGTTGGATGGAACTCCCAAAACCAGAAGC | ACGTTGGATGGAAGGAACTACCAAAACCTG | TGTTTCCTTCAAGGCAT |
| rs361525 | 6 | 31575324 | TNF | ACGTTGGATGAAGCATCAAGGATACCCCTC | ACGTTGGATGCACACAAATCAGTCAGTGGC | ACCCCCCTCGGAATC |
| rs187084 | 3 | 52227015 | TLR9 | ACGTTGGATGTATTCCCCTGCTGGAATGTC | ACGTTGGATGATGTGCTGGGCACTGTACTG | AAAAGATCACTGCCCT |
| rs1501299 | 3 | 186853334 | ADIPOQ | ACGTTGGATGCTCTTTCATCACAGACCTCC | ACGTTGGATGTCCCTGTGTCTAGGCCTTAG | GCTAGGCCTTAGTTAATAATGAATG |
| rs2571445 | 2 | 217818431 | TNS1 | ACGTTGGATGAACAGTGGGCACCAACACTC | ACGTTGGATGCAGCCATGCTGGGATTGATG | TCCCCGCTGGGATTGATGGCCCGCC |
| rs1051730 | 15 | 78601997 | CHRNA3 | ACGTTGGATGCAGCAGTTGTACTTGATGTC | ACGTTGGATGTCAAGGACTATTGGGAGAGC | CAAAGCCCCAGGCTA |
| rs153109 | 16 | 28507775 | IL27 | ACGTTGGATGTCAGTCAGTGACCAGGATCG | ACGTTGGATGATTCTTGGACCTGGTTGAGC | GCCTGACCTCACTCAACTC |
| rs12914385 | 15 | 78606381 | CHRNA3 | ACGTTGGATGGCTTGGTGGCTCTATTTTTG | ACGTTGGATGGCAAAAAAACAGAAGATGTC | CACAAGTAACTGAATTAACACACA |
| rs2245121 | 10 | 79939482 | SFTPD | ACGTTGGATGCACACACATCCAGATGAACC | ACGTTGGATGCATATGTGGGCATGCCTTTG | CAAGATATGTGCATCTACCCAAAGGTAC |
| rs207936 | 2 | 216175310 | XRCC5 | ACGTTGGATGAACTCTGGAAGTGGTGCAAG | ACGTTGGATGTCTACTTCTACCACCAGAGC | ACCCAGAGCCTCCTCTT |
| rs3785859 | 17 | 61302377 | BCAS3 | ACGTTGGATGCAGCAATAACTCATGTCCAC | ACGTTGGATGATTGGAATAGTGTGGTTAGC | TAGGGTGGTTAGCATGTTGAAGATTC |
| rs2672886 | 17 | 80807851 | RPTOR | ACGTTGGATGCATGTTTTCTTAAGGCCCCC | ACGTTGGATGACTTTGCAGGCCATGTAAGG | GCCATGTAAGGTCTCTG |
| rs2888674 | 7 | 150813827 | Intergenic | ACGTTGGATGACCATGCCAGCTTTATTCCC | ACGTTGGATGAGCAGGGAGTTAAACCAGAC | CCCCCCAGACTATGGCCCCA |
| rs17490056 | 13 | 66152771 | Intergenic | ACGTTGGATGTGTGGTATGACATCATAGGG | ACGTTGGATGGCAAATCTTTACCTTGGCCC | CCAGAAGAGGCCCAA |
| rs2241718 | 19 | 41323701 | CCDC97 | ACGTTGGATGCACAATTTTCCCTTCCTCCG | ACGTTGGATGGACTCAGGACCCATGATAAC | ACTAAGGACCCATGATAACAGCCTGTG |
| rs6830970 | 4 | 88855930 | FAM13A | ACGTTGGATGATCTGCCATGCTAAGTGGAC | ACGTTGGATGGGAGAAACCTTCTACTCCTC | GGATAAGCTGTCCAGTAAACGAAA |
| rs1800469 | 19 | 41354391 | TGFB1 | ACGTTGGATGAAGAGGGTCTGTCAACATGG | ACGTTGGATGCAATTCTTACAGGTGTCTGC | TGTATGCCTCCTGACCCTTCCATCC |
| rs10851906 | 15 | 78482334 | IREB2 | ACGTTGGATGGATCAGTCAGAATAAACAGGG | ACGTTGGATGCTGGCATTTCTATCGTTTCC | CCCCGCATTTTATGTGAATATAACCATC |
| rs3749893 | 6 | 116250532 | TSPYL4 | ACGTTGGATGCCTAAGTCCTTGAATTCACC | ACGTTGGATGGGTCACTCCTAGGATAATTG | GGGAGAGGATAATTGAGAGAAAACATC |
| rs2071278 | 6 | 32197667 | NOTCH4 | ACGTTGGATGTCTAAAGGACACAACAAGGG | ACGTTGGATGGAACTTAGCTTCCCAACACC | ACCCTCAGGTTTTGATTCTC |
| rs1800925 | 5 | 132657117 | IL-13 | ACGTTGGATGCAACACCCAACAGGCAAATG | ACGTTGGATGAGCCATGTCGCCTTTTCCTG | TCCTGCTCTTCCCTC |
| rs2282691 | 17 | 34361290 | CCL1 | ACGTTGGATGCATAGCTGTATCACAGGGTC | ACGTTGGATGCAAGAGGATCGACAGAGAGA | GGGAGAGCCTTAAAATACTGACTGGT |
| rs2227744 | 5 | 76714524 | F2R | ACGTTGGATGAGAGGCGATTTCGTTCTTCC | ACGTTGGATGGGTACTTCCGCAATTTAGTG | CCCACGCAATTTAGTGATCAACTTCT |
| rs652438 | 11 | 102865911 | MMP12 | ACGTTGGATGTCACAGATGACAAATACTGG | ACGTTGGATGCTCTTGGGATAATTTGGCTC | TAAGTTGGCTCTGGTCTTAAA |
| rs6751439 | 2 | 153874774 | GALNT13 | ACGTTGGATGATTGAGAACTAGTAGAAGGG | ACGTTGGATGCAGCCAAGTTGTAGACTTGC | CTACAAAGAAGCAGAAGAC |
| rs2878771 | 12 | 49958610 | AQP2 | ACGTTGGATGAGTATGGGACAGCTCAGAAC | ACGTTGGATGTCACACACACCAAACCAGAC | GGCCGCATGGAGACAGGTAGACA |
| rs1800796 | 7 | 22726627 | IL6 | ACGTTGGATGTCTTCTGTGTTCTGGCTCTC | ACGTTGGATGTGGAGACGCCTTGAAGTAAC | ATGCAGTTCTACAACAGCC |
| rs944899 | 13 | 112096647 | SOX1-OT | ACGTTGGATGCATAGGAAGTCCAGTTGGTG | ACGTTGGATGGGCTCACAAAGGCCTTTTAC | GATGCTCCCCACACAATGAACATGCT |
| rs2280090 | 20 | 3669558 | ADAM33 | ACGTTGGATGATGAGCCCTTCCCTTCTCC | ACGTTGGATGCGTTCACCCCATGGAGTTG | CTTTTCCCCACAGCCACTGGACAG |
| rs2077079 | 2 | 85668215 | SFTPB | ACGTTGGATGAAGACAAACACTGAGGTCGC | ACGTTGGATGTGACTCAGCCATGGCACCT | GGGTACCCTGCTTGG |
| rs1801270 | 6 | 36684194 | CDKN1A | ACGTTGGATGCCGCCATTAGCGCATCACA | ACGTTGGATGATGTCCGTCAGAACCCATGC | GGACCAGTGGACAGCGAGCAGCTGAG |
| rs2364723 | 2 | 177261818 | NFE2L2 | ACGTTGGATGTTCCTCTGTCCTGACTGAAG | ACGTTGGATGTTAACCCAGGCTTGAGGAAC | GGGTGGGCTTGAGGAACAGTTAA |
| rs6574978 | 14 | 88011069 | GPR65 | ACGTTGGATGAAGCACTCCCTTTGCACAAG | ACGTTGGATGCTCTATGCATTAACTCTCCC | CGCTCCCTTTATGGATTGATTATAC |
| rs3025030 | 6 | 43782850 | VEGFA | ACGTTGGATGTGGCTGTTCGTTTAGGATGG | ACGTTGGATGGACTGGTGGAGGATTAAAGG | GGAGGATTAAAGGTATCTAGTATT |
| rs28929474 | 14 | 94378610 | *SERPINA1* | ACGTTGGATGGTGCATAAGGCTGTGCTGAC | ACGTTGGATGATAGACATGGGTATGGCCTC | AGCTTCAGTCCCTTTCT |
| rs7326277 | 13 | 28302077 | *FLT1* | ACGTTGGATGATCAAAACGAGGGCTGATGG | ACGTTGGATGTGGTGAATTGGTTTGGTTGG | CCTGGGTATAGAGACGGGGT |
| rs1800795 | 7 | 22727026 | *IL6* | ACGTTGGATGAGCCTCAATGACGACCTAAG | ACGTTGGATGGATTGTGCAATGTGACGTCC | ACTGTGACGTCCTTTAGCAT |
| rs16969968 | 15 | 78590583 | CHRNA5 | ACGTTGGATGAGTGGTAGTGGACCAAAATC | ACGTTGGATGACCTCACGGACATCATTTTC | GTCTTGTAATGTAGCGAATAGAAT |
| rs59569785 | 12 | 23618801 | SOX5 | ACGTTGGATGCCTCTATCCCACTGCAAAAG | ACGTTGGATGTCCAAGTGGCCCTGAAATTC | GTAATGCTACTATGGGAAAA |

SNPs: Single nucleotide polymorphisms; Chr: Chromosome; UEP_SEQ: unextended minisequencing primer;
