# Supplementary material for: Comparison and development of machine learning tools for the prediction of chronic obstructive pulmonary disease in the Chinese population
Source: J Transl Med. 2020 Mar 31;18:146. doi: 10.1186/s12967-020-02312-0 (PMC7110698; doi:10.1186/s12967-020-02312-0)
Supplement: Supplementary file 5 — Additional file 5: Table S5. The parameters selection in the predictive models. [file 12967_2020_2312_MOESM5_ESM.docx]

Additional file 5: Table S5 The parameters selection in the predictive models

| **Models** | **Tuning parameter** | **Models** | **default Parameter** |
| --- | --- | --- | --- |
| LR | Penalty = 'l1'，  C (Cost) =10 ,  max_iter=50,  intercept_scaling=10 | KNN | K (#Neighbors)=5 |
| MLP | α(Regularization parameter) hidden_layer_size = (8,8,8) | SVM | α (Regularization parameter)=1 |
| XGboost | scale_pos_weight=0.08  learning_rate=0.05  n_estimators=1000, max_depth =10,  reg_alpha=0.2,  subsample=1 | DT | min_samples_split=2 |

KNN: k-nearest neighbors classifier; LR: logistic regression; SVM: support vector machine; DT: decision tree; MLP: multilayer perceptron.
