# Supplementary material for: Comparison and development of machine learning tools for the prediction of chronic obstructive pulmonary disease in the Chinese population
Source: J Transl Med. 2020 Mar 31;18:146. doi: 10.1186/s12967-020-02312-0 (PMC7110698; doi:10.1186/s12967-020-02312-0)
Supplement: Supplementary file 6 — Additional file 6: Table S6. Allele frequencies in COPD and control subjects for SNPs. [file 12967_2020_2312_MOESM6_ESM.docx]

**Additional file 6: Table S6 Allele frequencies in COPD and control subjects for SNPs**

| **CHR** | **SNPs** | **position** | **A1** | **F_A** | **F_U** | **A2** | **CHISQ** | **P value** | **OR** | **OR 95%（CI)** |
| --- | --- | --- | --- | --- | --- | --- | --- | --- | --- | --- |
| 4 | **rs10007052** | 141084419 | C | 0.288 | 0.195 | A | 6.633 | **0.010** | **1.671** | 1.128-2.477 |
| 4 | **rs8192288** | 24795056 | T | 0.126 | 0.196 | G | 5.939 | **0.015** | **0.593** | 0.388-0.906 |
| 5 | **rs20541** | 132660272 | A | 0.305 | 0.396 | G | 5.607 | **0.018** | **0.670** | 0.480-0.934 |
| 16 | **rs12922394** | 82638722 | T | 0.198 | 0.275 | C | 5.228 | **0.022** | **0.651** | 0.449-0.942 |
| 5 | **rs2910164** | 160485411 | G | 0.452 | 0.368 | C | 4.332 | **0.037** | **1.416** | 1.020-1.967 |
| 12 | **rs161976** | 32215232 | A | 0.211 | 0.144 | G | 4.061 | **0.044** | **1.594** | 1.010-2.515 |
| 6 | **rs473892** | 137629910 | C | 0.286 | 0.214 | T | 4.032 | **0.045** | **1.473** | 1.008-2.152 |
| 5 | **rs159497** | 58912391 | T | 0.346 | 0.268 | C | 4.018 | **0.045** | **1.445** | 1.007-2.073 |
| 6 | **rs9296092** | 33510719 | A | 0.118 | 0.068 | G | 4.009 | **0.045** | **1.832** | 1.005-3.340 |
| 12 | rs1690139 | 75878897 | C | 0.390 | 0.314 | T | 3.571 | 0.059 | 1.395 | 0.987-1.973 |
| 16 | rs4148382 | 16144637 | A | 0.056 | 0.091 | G | 2.749 | 0.097 | 0.591 | 0.315-1.107 |
| 2 | rs3024791 | 85668581 | T | 0.029 | 0.054 | C | 2.667 | 0.103 | 0.516 | 0.229-1.157 |
| 4 | rs4073 | 73740307 | A | 0.415 | 0.349 | T | 2.573 | 0.109 | 1.321 | 0.939-1.858 |
| 6 | rs915895 | 32222440 | T | 0.489 | 0.427 | C | 2.341 | 0.126 | 1.287 | 0.931-1.780 |
| 4 | rs1542725 | 144567182 | T | 0.306 | 0.364 | C | 2.330 | 0.127 | 0.771 | 0.552-1.077 |
| 5 | rs10461985 | 37874307 | A | 0.129 | 0.090 | G | 2.144 | 0.143 | 1.498 | 0.869-2.580 |
| 4 | rs17050782 | 139501980 | G | 0.372 | 0.316 | A | 2.141 | 0.143 | 1.287 | 0.918-1.806 |
| 2 | rs1130866 | 85666618 | A | 0.258 | 0.209 | G | 1.867 | 0.172 | 1.313 | 0.888-1.942 |
| 6 | rs1800630 | 31574699 | A | 0.176 | 0.136 | C | 1.757 | 0.185 | 1.357 | 0.863-2.132 |
| 16 | rs181206 | 28502082 | G | 0.166 | 0.129 | A | 1.532 | 0.216 | 1.342 | 0.841-2.142 |
| 3 | rs1801282 | 12351626 | G | 0.079 | 0.053 | C | 1.517 | 0.218 | 1.527 | 0.775-3.008 |
| 2 | rs11677877 | 227266453 | G | 0.185 | 0.147 | A | 1.476 | 0.224 | 1.314 | 0.8451-2.042 |
| 10 | rs911887 | 79941767 | C | 0.392 | 0.345 | T | 1.466 | 0.226 | 1.228 | 0.881-1.712 |
| 23 | rs6609533 | 47585887 | G | 0.477 | 0.536 | A | 1.394 | 0.238 | 0.788 | 0.530-1.171 |
| 3 | rs2243115 | 159988493 | G | 0.090 | 0.117 | T | 1.222 | 0.269 | 0.750 | 0.449-1.251 |
| 1 | rs1051740 | 225831932 | C | 0.360 | 0.403 | T | 1.206 | 0.272 | 0.833 | 0.6012-1.154 |
| 4 | rs7041 | 71752617 | C | 0.261 | 0.301 | A | 1.172 | 0.279 | 0.818 | 0.5678-1.178 |
| 2 | rs231775 | 203867991 | A | 0.336 | 0.295 | G | 1.118 | 0.290 | 1.207 | 0.851-1.712 |
| 15 | rs8034191 | 78513681 | C | 0.040 | 0.025 | T | 1.081 | 0.298 | 1.673 | 0.628-4.462 |
| 19 | rs3733829 | 40804666 | G | 0.394 | 0.352 | A | 1.064 | 0.302 | 1.195 | 0.852-1.676 |
| 1 | rs2234922 | 225838705 | G | 0.107 | 0.132 | A | 0.957 | 0.328 | 0.786 | 0.484-1.275 |
| 6 | rs2070600 | 32183666 | T | 0.212 | 0.245 | C | 0.918 | 0.338 | 0.830 | 0.567-1.215 |
| 16 | rs35621 | 16074751 | T | 0.281 | 0.317 | C | 0.883 | 0.348 | 0.842 | 0.588-1.206 |
| 5 | rs25882 | 132075767 | T | 0.431 | 0.393 | C | 0.869 | 0.351 | 1.167 | 0.843-1.614 |
| 1 | rs17111652 | 55124792 | T | 0.055 | 0.073 | C | 0.842 | 0.359 | 0.744 | 0.394-1.403 |
| 11 | rs2276109 | 102875061 | C | 0.026 | 0.015 | T | 0.808 | 0.369 | 1.761 | 0.505-6.147 |
| 5 | rs10473352 | 44308150 | G | 0.183 | 0.155 | A | 0.790 | 0.374 | 1.216 | 0.789-1.873 |
| 5 | rs1011814 | 44335718 | C | 0.495 | 0.460 | T | 0.719 | 0.396 | 1.150 | 0.833-1.587 |
| 4 | rs4863687 | 139757127 | T | 0.243 | 0.273 | C | 0.694 | 0.405 | 0.856 | 0.593-1.235 |
| 19 | rs2604894 | 40786499 | A | 0.293 | 0.324 | G | 0.664 | 0.415 | 0.867 | 0.615-1.222 |
| 3 | rs9863587 | 197854276 | A | 0.504 | 0.471 | G | 0.652 | 0.420 | 1.141 | 0.829-1.57 |
| 2 | rs734556 | 223696612 | T | 0.220 | 0.193 | C | 0.630 | 0.427 | 1.181 | 0.783-1.779 |
| 20 | rs528557 | 3671095 | G | 0.226 | 0.199 | C | 0.610 | 0.435 | 1.173 | 0.785-1.753 |
| 17 | rs2020936 | 30223796 | G | 0.116 | 0.136 | A | 0.576 | 0.448 | 0.833 | 0.518-1.337 |
| 5 | rs1042713 | 148826877 | G | 0.426 | 0.396 | A | 0.548 | 0.459 | 1.134 | 0.813-1.583 |
| 3 | rs6577641 | 18356357 | C | 0.068 | 0.054 | T | 0.523 | 0.470 | 1.289 | 0.647-2.567 |
| 8 | rs11779254 | 4391552 | C | 0.451 | 0.481 | G | 0.521 | 0.471 | 0.889 | 0.647-1.223 |
| 15 | rs660652 | 78595490 | A | 0.214 | 0.190 | G | 0.520 | 0.471 | 1.161 | 0.774-1.742 |
| 5 | rs16878037 | 60413925 | T | 0.131 | 0.112 | C | 0.499 | 0.480 | 1.196 | 0.728-1.966 |
| 5 | rs3995090 | 148466252 | A | 0.241 | 0.265 | C | 0.449 | 0.503 | 0.882 | 0.611-1.274 |
| 1 | rs584367 | 20115561 | T | 0.264 | 0.240 | C | 0.448 | 0.503 | 1.135 | 0.783-1.646 |
| 20 | rs17576 | 46011586 | A | 0.275 | 0.299 | G | 0.441 | 0.507 | 0.887 | 0.623-1.263 |
| 12 | rs10859974 | 95895082 | C | 0.416 | 0.442 | T | 0.429 | 0.513 | 0.898 | 0.652-1.238 |
| 15 | rs7181486 | 78449276 | C | 0.130 | 0.113 | T | 0.420 | 0.517 | 1.179 | 0.717-1.940 |
| 8 | rs13278529 | 40475802 | G | 0.055 | 0.044 | T | 0.405 | 0.524 | 1.278 | 0.599-2.726 |
| 4 | rs1903003 | 88965146 | C | 0.402 | 0.376 | T | 0.379 | 0.538 | 1.113 | 0.792-1.565 |
| 3 | rs3773445 | 25571074 | G | 0.461 | 0.485 | A | 0.363 | 0.547 | 0.906 | 0.657-1.249 |
| 10 | rs3088308 | 79938112 | T | 0.014 | 0.019 | A | 0.320 | 0.572 | 0.706 | 0.210-2.371 |
| 15 | rs10519225 | 49428581 | A | 0.176 | 0.158 | G | 0.315 | 0.575 | 1.135 | 0.729-1.765 |
| 4 | rs2202507 | 144336529 | G | 0.427 | 0.404 | T | 0.311 | 0.577 | 1.098 | 0.789-1.527 |
| 10 | rs701848 | 87966988 | C | 0.445 | 0.422 | T | 0.310 | 0.578 | 1.096 | 0.794-1.513 |
| 6 | rs3025033 | 43783338 | G | 0.186 | 0.170 | A | 0.272 | 0.602 | 1.118 | 0.735-1.700 |
| 2 | rs6435156 | 202560752 | T | 0.090 | 0.078 | C | 0.266 | 0.606 | 1.166 | 0.649-2.092 |
| 6 | rs361525 | 31575324 | A | 0.047 | 0.039 | G | 0.223 | 0.637 | 1.214 | 0.542-2.717 |
| 3 | rs187084 | 52227015 | G | 0.436 | 0.418 | A | 0.217 | 0.641 | 1.080 | 0.783-1.490 |
| 3 | rs1501299 | 186853334 | T | 0.280 | 0.263 | G | 0.205 | 0.651 | 1.090 | 0.752-1.580 |
| 2 | rs2571445 | 217818431 | A | 0.424 | 0.406 | G | 0.198 | 0.657 | 1.077 | 0.777-1.491 |
| 15 | rs1051730 | 78601997 | A | 0.019 | 0.015 | G | 0.196 | 0.658 | 1.336 | 0.369-4.838 |
| 16 | rs153109 | 28507775 | C | 0.389 | 0.407 | T | 0.195 | 0.659 | 0.929 | 0.671-1.287 |
| 15 | rs12914385 | 78606381 | T | 0.286 | 0.272 | C | 0.142 | 0.706 | 1.072 | 0.747-1.537 |
| 10 | rs2245121 | 79939482 | G | 0.257 | 0.269 | A | 0.100 | 0.752 | 0.941 | 0.647-1.370 |
| 2 | rs207936 | 216175310 | T | 0.059 | 0.064 | C | 0.087 | 0.768 | 0.905 | 0.468-1.752 |
| 17 | rs3785859 | 61302377 | A | 0.266 | 0.255 | C | 0.081 | 0.776 | 1.056 | 0.725-1.539 |
| 17 | rs2672886 | 80807851 | C | 0.451 | 0.440 | T | 0.073 | 0.787 | 1.046 | 0.756-1.446 |
| 7 | rs2888674 | 150813827 | G | 0.229 | 0.238 | A | 0.063 | 0.803 | 0.953 | 0.656-1.387 |
| 13 | rs17490056 | 66152771 | C | 0.363 | 0.354 | T | 0.060 | 0.806 | 1.043 | 0.744-1.464 |
| 19 | rs2241718 | 41323701 | A | 0.302 | 0.311 | G | 0.047 | 0.828 | 0.961 | 0.673-1.372 |
| 4 | rs6830970 | 88855930 | A | 0.459 | 0.451 | G | 0.035 | 0.852 | 1.033 | 0.738-1.445 |
| 19 | rs1800469 | 41354391 | G | 0.488 | 0.495 | A | 0.027 | 0.870 | 0.973 | 0.699-1.352 |
| 15 | rs10851906 | 78482334 | G | 0.245 | 0.250 | A | 0.022 | 0.883 | 0.972 | 0.663-1.424 |
| 6 | rs3749893 | 116250532 | G | 0.349 | 0.354 | A | 0.020 | 0.889 | 0.977 | 0.700-1.362 |
| 6 | rs2071278 | 32197667 | G | 0.174 | 0.179 | A | 0.018 | 0.892 | 0.971 | 0.635-1.485 |
| 5 | rs1800925 | 132657117 | T | 0.159 | 0.162 | C | 0.009 | 0.926 | 0.979 | 0.625-1.533 |
| 17 | rs2282691 | 34361290 | T | 0.356 | 0.360 | A | 0.008 | 0.928 | 0.984 | 0.691-1.401 |
| 5 | rs2227744 | 76714524 | A | 0.275 | 0.272 | G | 0.006 | 0.939 | 1.014 | 0.708-1.453 |
| 11 | rs652438 | 102865911 | C | 0.082 | 0.084 | T | 0.006 | 0.940 | 0.977 | 0.540-1.768 |
| 2 | rs6751439 | 153874774 | G | 0.143 | 0.145 | A | 0.004 | 0.948 | 0.985 | 0.623-1.558 |
| 12 | rs2878771 | 49958610 | C | 0.407 | 0.404 | G | 0.004 | 0.948 | 1.011 | 0.727-1.406 |
| 7 | rs1800796 | 22726627 | G | 0.362 | 0.364 | C | 0.003 | 0.959 | 0.991 | 0.712-1.380 |
| 13 | rs944899 | 112096647 | A | 0.488 | 0.490 | G | 0.002 | 0.965 | 0.993 | 0.716-1.377 |
| 20 | rs2280090 | 3669558 | A | 0.069 | 0.070 | G | 0.002 | 0.965 | 0.986 | 0.522-1.861 |
| 2 | rs2077079 | 85668215 | T | 0.489 | 0.490 | G | 0.001 | 0.980 | 0.996 | 0.724-1.371 |
| 6 | rs1801270 | 36684194 | A | 0.438 | 0.437 | C | 0.000 | 0.988 | 1.002 | 0.727-1.382 |
| 2 | rs2364723 | 177261818 | G | 0.495 | 0.495 | C | 0.000 | 0.996 | 0.999 | 0.724-1.378 |
| 14 | rs6574978 | 88011069 | C | 0.029 | 0.029 | T | 0.000 | 1.000 | 1.000 | 0.389-2.572 |

CHR: Chromosome; SNPs: Single nucleotide polymorphisms; A1: minor Allele; A2: main Allele; F_A: Frequency-Affect (A1 ratio); F_U: Frequency-UnAffect (A1 ratio); OR: Odds ratio; 95%(CI): 95% confidence interval.
