# Supplementary figures and images for: Comparison and development of machine learning tools for the prediction of chronic obstructive pulmonary disease in the Chinese population
Source: J Transl Med. 2020 Mar 31;18:146. doi: 10.1186/s12967-020-02312-0 (PMC7110698; doi:10.1186/s12967-020-02312-0)

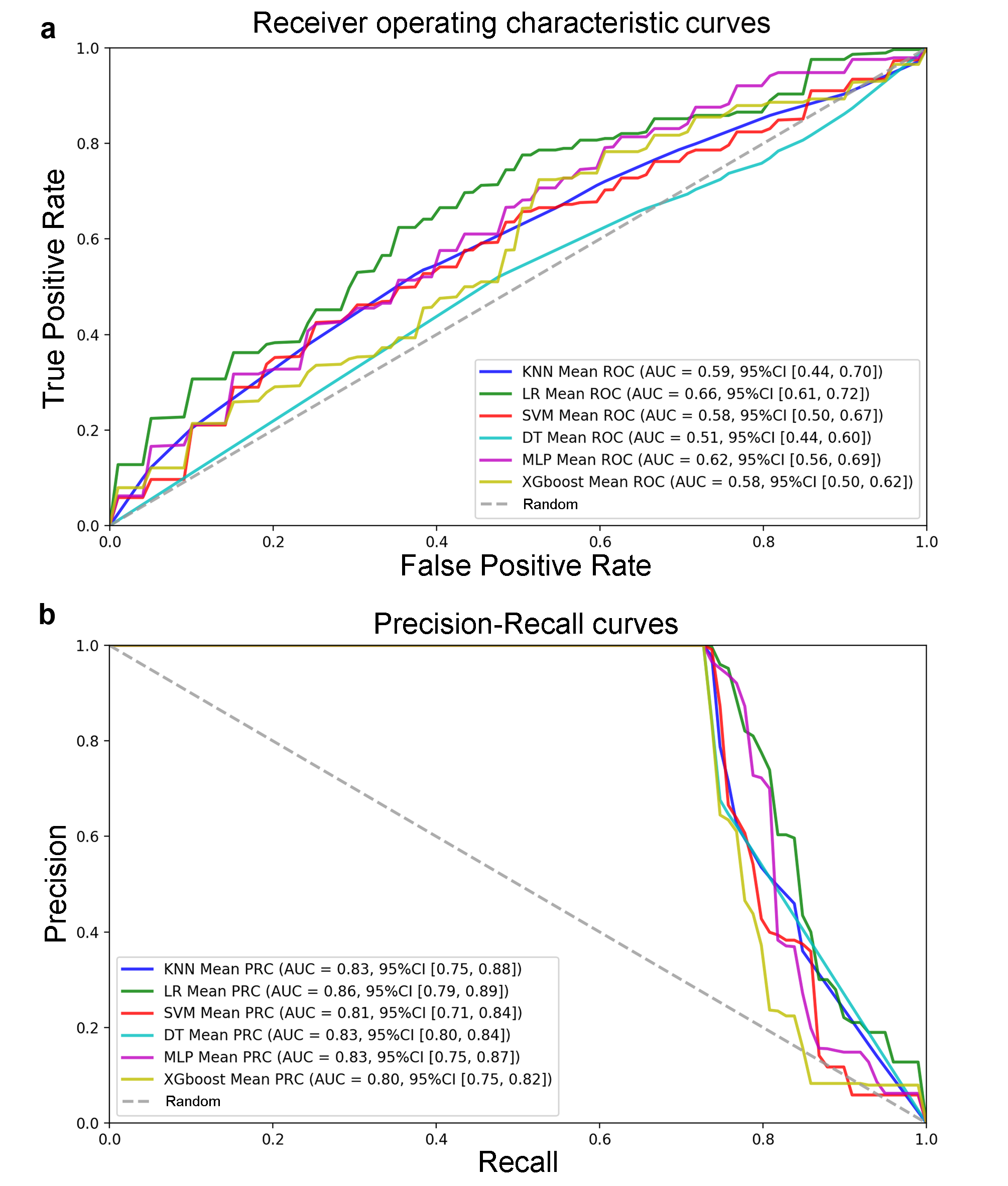

Supplement: Supplementary file 7 — Additional file 7: Fig. S1. Evaluation of the predictive models with only the 9 SNPs as inputs. a, b The picture shows the AU-ROC and AU-PRC curves of the 6 models in the training set. Mean AUC values and 95% CIs of different prediction models are shown in the box. [file 12967_2020_2312_MOESM7_ESM.tif]

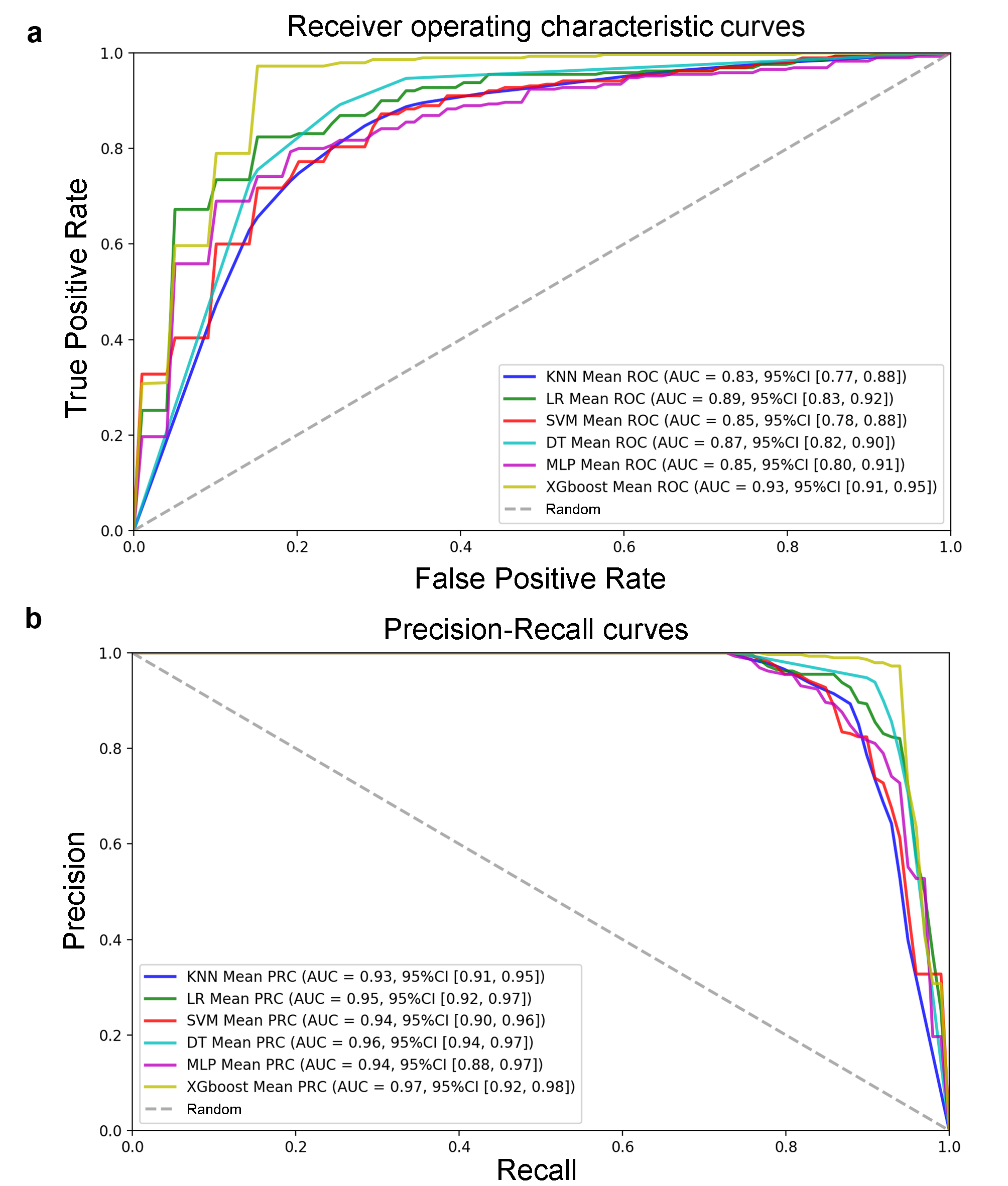

Supplement: Supplementary file 9 — Additional file 9: Fig. S2. Evaluation of the predictive models with only the 5 clinical features as inputs. a, b The picture shows the AU-ROC and AU-PRC curves of the 6 models in the training set. Mean AUC values and 95% CIs of different prediction models are shown in the box. [file 12967_2020_2312_MOESM9_ESM.tif]

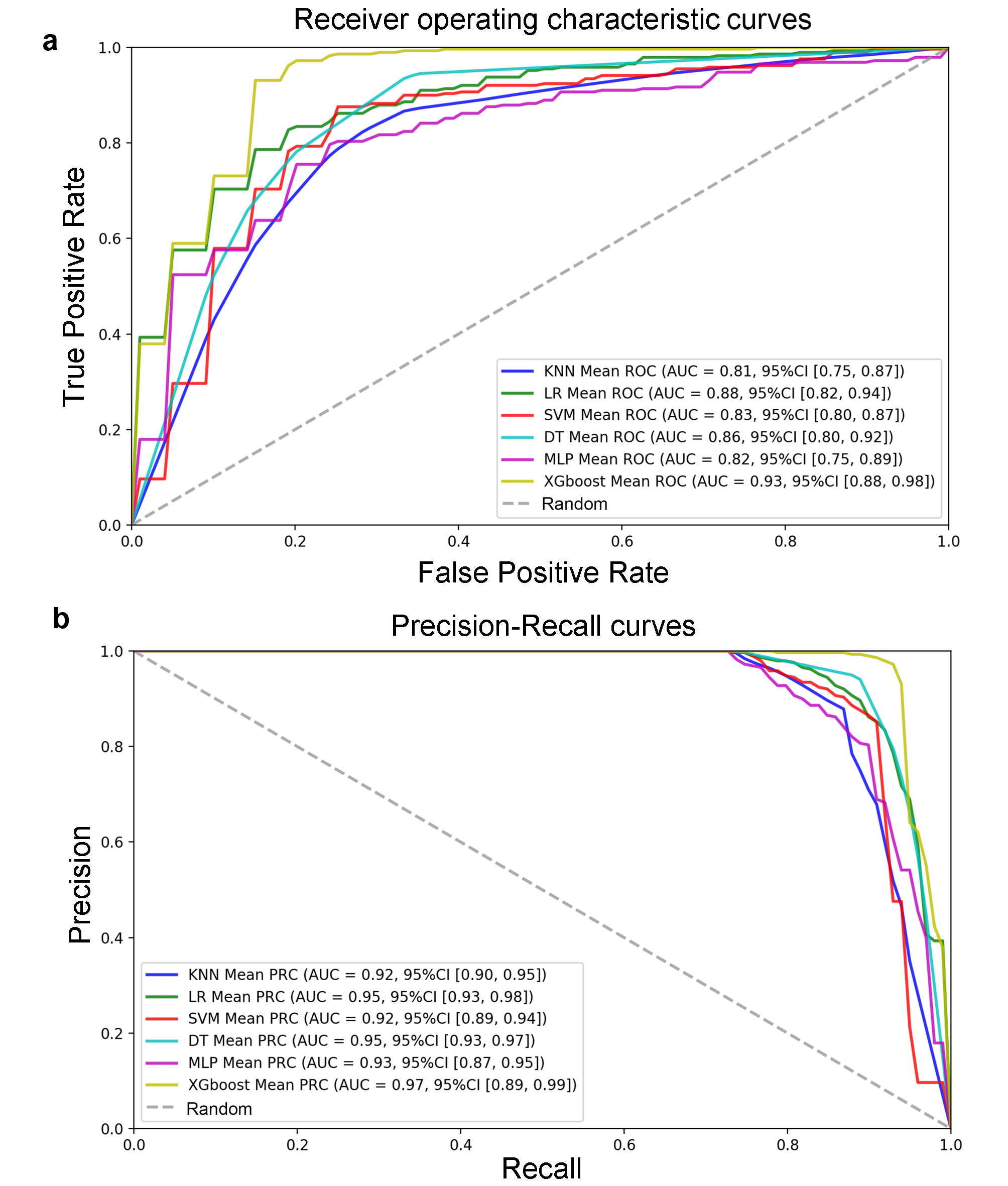

Supplement: Supplementary file 11 — Additional file 11: Fig. S3. Evaluation of the predictive models with the top 5 ranked features as inputs. a, b The picture shows the AU-ROC and AU-PRC curves of the 6 models in the training set. Mean AUC values and 95% CIs of different prediction models are shown in the box. [file 12967_2020_2312_MOESM11_ESM.tif]

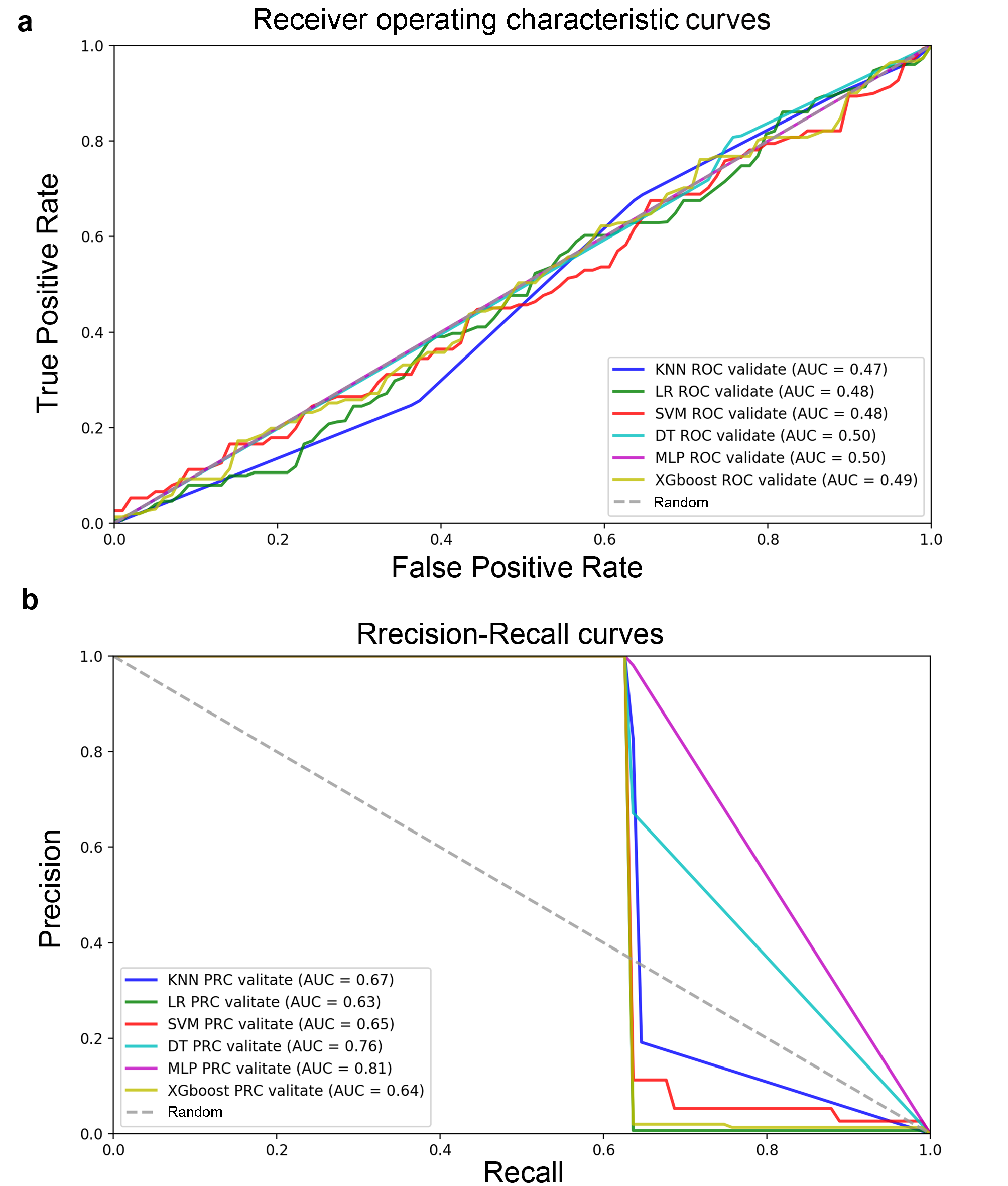

Supplement: Supplementary file 14 — Additional file 14: Fig. S4. Validation of the models in the training set with only the 9 SNPs as inputs. a, b The picture shows the AU-ROC and AU-PRC curves of all models in the test set. [file 12967_2020_2312_MOESM14_ESM.tif]

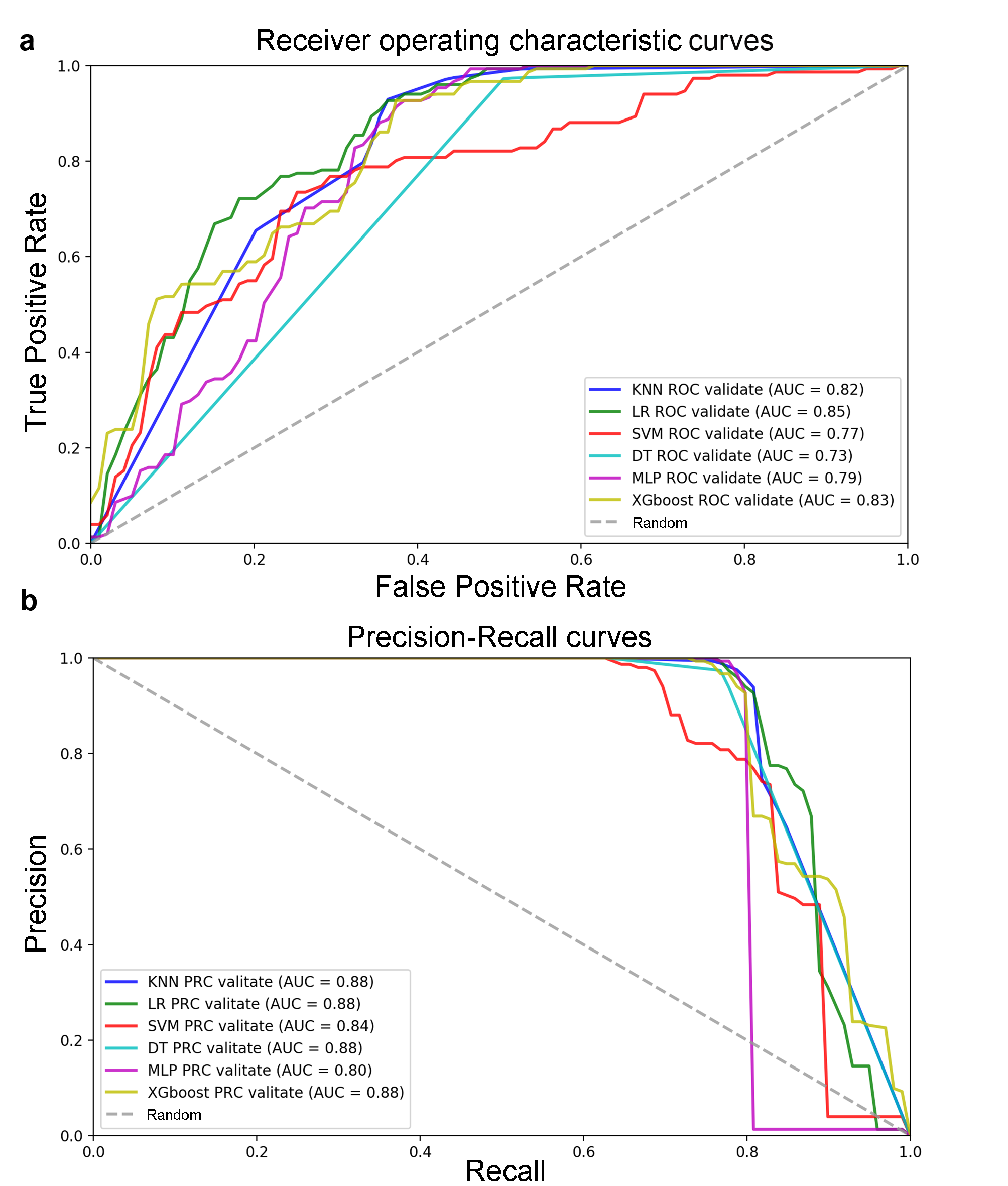

Supplement: Supplementary file 15 — Additional file 15: Fig. S5. Validation of the models in the training set with only the 5 clinical features as inputs. a, b The picture shows the AU-ROC and AU-PRC curves of all models in the test set. [file 12967_2020_2312_MOESM15_ESM.tif]

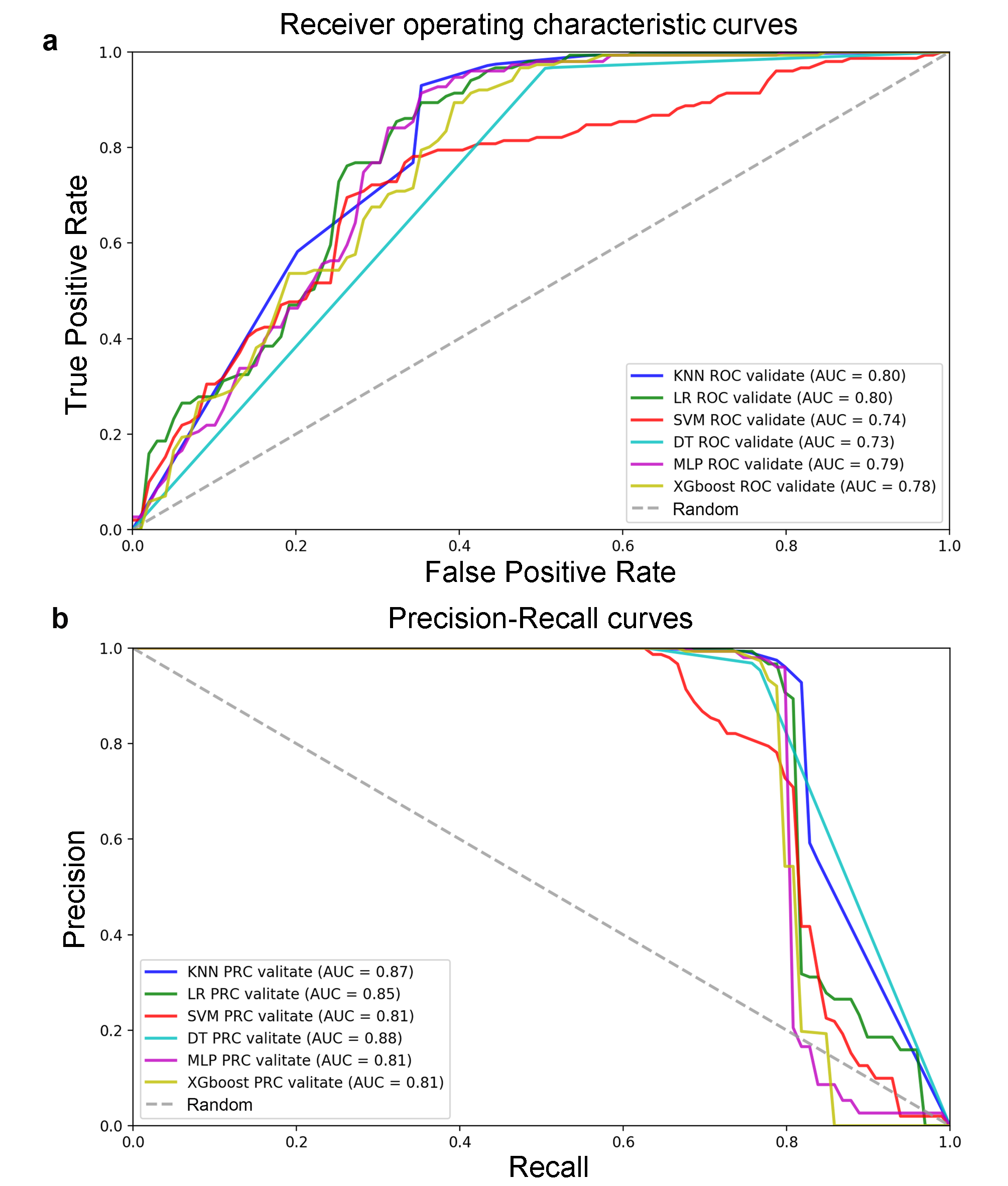

Supplement: Supplementary file 17 — Additional file 17: Fig. S6. Validation of the models in the training set with the top 5 ranked features as inputs. a, b The picture shows the AU-ROC and AU-PRC curves of all models in the test set. [file 12967_2020_2312_MOESM17_ESM.tif]
