# Supplementary material for: Comparison and development of machine learning tools for the prediction of chronic obstructive pulmonary disease in the Chinese population
Source: J Transl Med. 2020 Mar 31;18:146. doi: 10.1186/s12967-020-02312-0 (PMC7110698; doi:10.1186/s12967-020-02312-0)
Supplement: Supplementary file 8 — Additional file 8: Table S7. The efficacy of KNN, LR, SVM, DT, MLP and XGboost in the training set of 9 SNPs features. [file 12967_2020_2312_MOESM8_ESM.docx]

**Additional file 8: Table S7 The efficacy of KNN, LR, SVM, DT, MLP and XGboost in the training set of 9 SNPs features**

| **Metrics** | **KNN** | **LR** | **SVM** | **DT** | **MLP** | **XGboost** |
| --- | --- | --- | --- | --- | --- | --- |
|  | **(95%CI)** | **(95%CI)** | **(95%CI)** | **(95%CI)** | **(95%CI)** | **(95%CI)** |
| AU-ROC | 0.59(0.44-0.70) | 0.66(0.61-0.72) | 0.58(0.50-0.67) | 0.51(0.44-0.60) | 0.62(0.56-0.69) | 0.58(0.50-0.62) |
| AU-PRC | 0.83(0.75-0.88) | 0.86 (0.79-0.89) | 0.81(0.71-0.84) | 0.83(0.80-0.84) | 0.83(0.75-0.87) | 0.80(0.75-0.82) |
| accuracy | 0.68(0.60-0.74) | 0.74(0.71-0.79) | 0.74(0.73-0.74) | 0.62(0.53-0.68) | 0.74(0.73-0.74) | 0.64(0.58-0.68) |
| precision | 0.76(0.71-0.79) | 0.76(0.74-0.79) | 0.74(0.73-0.74) | 0.74(0.70-0.81) | 0.74(0.73-0.74) | 0.78(0.75-0.80) |
| recall | 0.83(0.76-0.89) | 0.95(0.92-0.98) | 1.00(1.00-1.00) | 0.72(0.62-0.79) | 1.00(1.00-1.00) | 0.71(0.61-0.81) |
| F1 score | 0.79(0.74-0.84) | 0.84(0.82-0.87) | 0.85(0.84-0.85) | 0.74(0.66-0.78) | 0.85(0.84-0.85） | 0.74(0.68-0.79) |
| MCC | 0.10(-0.13-0.26) | 0.17(0.08-0.36) | 0 | -0.10(-0.13-0.10) | 0 | 0.13(0.07-0.21) |
| SPC | 0.24(0.11-0.33) | 0.16(0.10-0.024) | 0 | 0.33(0.20-0.51) | 0 | 0.42(0.29-0.52) |
| NPV | 0.36(0.15-0.53) | 0.53(0.38-0.81) | *NA* | 0.28(0.20-0.41) | *NA* | 0.34(0.30-0.39) |

AU-ROC, area under the receiver operating characteristic curve; AU-PRC, area under the precision-recall curve; MCC, Matthews correlation coefficient; SPC, specificity; NPV, negative prognostic value; KNN, k-nearest neighbors classifier; LR, logistic regression; SVM, support vector machine; DT, decision tree; MLP, multilayer perceptron; 95%CI, 95% confidence interval; *NA*: not available.
