# Supplementary material for: Comparison and development of machine learning tools for the prediction of chronic obstructive pulmonary disease in the Chinese population
Source: J Transl Med. 2020 Mar 31;18:146. doi: 10.1186/s12967-020-02312-0 (PMC7110698; doi:10.1186/s12967-020-02312-0)
Supplement: Supplementary file 12 — Additional file 12: Table S9. The efficacy of KNN, LR, SVM, DT, MLP and XGboost in the training set of top 5 ranked features. [file 12967_2020_2312_MOESM12_ESM.docx]

**Additional file 12: Table S9 The efficacy of KNN, LR, SVM, DT, MLP and XGboost in the training set of top 5 ranked features**

| **Metrics** | **KNN** | **LR** | **SVM** | **DT** | **MLP** | **XGboost** |
| --- | --- | --- | --- | --- | --- | --- |
|  | **(95%CI)** | **(95%CI)** | **(95%CI)** | **(95%CI)** | **(95%CI)** | **(95%CI)** |
| AU-ROC | 0.81(0.75-0.87) | 0.88(0.82-0.94) | 0.83(0.80-0.87) | 0.86(0.80-0.92) | 0.82(0.75-0.89) | 0.93(0.88-0.98) |
| AU-PRC | 0.92(0.90-0.95) | 0.95(0.93-0.98) | 0.92(0.89-0.94) | 0.95(0.93-0.97) | 0.93(0.87-0.95) | 0.97(0.89-0.99) |
| accuracy | 0.82(0.75-0.86） | 0.84(0.76-0.89) | 0.80(0.77-0.83) | 0.89(0.86-0.93) | 0.79(0.75-0.85) | 0.91(0.88-0.96) |
| precision | 0.87(0.82-0.91) | 0.86(0.83-0.90) | 0.83(0.80-0.85) | 0.92(0.89-0.96) | 0.83(0.78-0.90) | 0.94(0.92-0.97) |
| recall | 0.89(0.85-0.91) | 0.93(0.85-1.00) | 0.92(0.91-0.95) | 0.93(0.91-0.96) | 0.90(0.82-0.99) | 0.94(0.90-0.98) |
| F1 score | 0.88(0.84-0.90) | 0.89(0.84-0.93) | 0.87(0.86-0.89) | 0.93(0.91-0.95) | 0.86(0.83-0.91) | 0.94(0.91-0.97) |
| MCC | 0.53(0.35-0.63) | 0.57(0.39-0.72) | 0.45(0.34-0.53) | 0.72(0.64-0.83) | 0.43(0.26-0.61) | 0.78(0.69-0.89) |
| SPC | 0.63(0.48-0.76) | 0.58(0.48-0.69) | 0.47(0.34-0.57) | 0.78(0.65-0.90） | 0.49(0.30-0.74) | 0.84(0.76-0.900 |
| NPV | 0.66(0.54-0.72) | 0.79(0.56-1.00) | 0.68(0.62-0.76) | 0.81(0.76-0.87) | 0.67(0.53-0.97) | 0.84(0.74-0.95) |

AU-ROC, area under the receiver operating characteristic curve; AU-PRC, area under the precision-recall curve; MCC, Matthews correlation coefficient; SPC, specificity; NPV, negative prognostic value; KNN, k-nearest neighbors classifier; LR, logistic regression; SVM, support vector machine; DT, decision tree; MLP, multilayer perceptron; 95%CI, 95% confidence interval.
