# Supplementary material for: Comparison and development of machine learning tools for the prediction of chronic obstructive pulmonary disease in the Chinese population
Source: J Transl Med. 2020 Mar 31;18:146. doi: 10.1186/s12967-020-02312-0 (PMC7110698; doi:10.1186/s12967-020-02312-0)
Supplement: Supplementary file 13 — Additional file 13: Table S10. The efficacy of KNN, LR, SVM, DT, MLP and XGboost in the test set of 9 SNPs features. [file 12967_2020_2312_MOESM13_ESM.docx]

**Additional file 13: Table S10 The efficacy of KNN, LR, SVM, DT, MLP and XGboost in the test set of 9 SNPs features**

| **Metrics** | **KNN** | **LR** | **SVM** | **DT** | **MLP** | **XGboost** |
| --- | --- | --- | --- | --- | --- | --- |
| AU-ROC | 0.47 | 0.48 | 0.48 | 0.50 | 0.50 | 0.49 |
| AU-PRC | 0.67 | 0.63 | 0.65 | 0.76 | 0.81 | 0.64 |
| accuracy | 0.61 | 0.61 | 0.63 | 0.55 | 0.63 | 0.48 |
| precision | 0.63 | 0.62 | 0.63 | 0.62 | 0.63 | 0.63 |
| recall | 0.90 | 0.97 | 1.00 | 0.72 | 1.00 | 0.42 |
| F1 score | 0.74 | 0.76 | 0.77 | 0.67 | 0.77 | 0.51 |
| MCC | 0.02 | -0.07 | 0 | -0.02 | 0 | 0 |
| SPC | 0.11 | 0.01 | 0 | 0.27 | 0 | 0.57 |
| NPV | 0.40 | 0.17 | *NA* | 0.36 | *NA* | 0.37 |

AU-ROC, area under the receiver operating characteristic curve; AU-PRC, area under the precision-recall curve; MCC, Matthews correlation coefficient; SPC, specificity; NPV, negative prognostic value; KNN, k-nearest neighbors classifier; LR, logistic regression; SVM, support vector machine; DT, decision tree; MLP, multilayer perceptron; *NA: not available.*
