# Supplementary material for: Comparison and development of machine learning tools for the prediction of chronic obstructive pulmonary disease in the Chinese population
Source: J Transl Med. 2020 Mar 31;18:146. doi: 10.1186/s12967-020-02312-0 (PMC7110698; doi:10.1186/s12967-020-02312-0)
Supplement: Supplementary file 16 — Additional file 16: Table S11. The efficacy of KNN, LR, SVM, DT, MLP and XGboost in the test set of 5 clinical features. [file 12967_2020_2312_MOESM16_ESM.docx]

**Additional file 16: Table S11 The efficacy of KNN, LR, SVM, DT, MLP and XGboost in the test set of 5 clinical features**

| **Metrics** | **KNN** | **LR** | **SVM** | **DT** | **MLP** | **XGboost** |
| --- | --- | --- | --- | --- | --- | --- |
| AU-ROC | 0.82 | 0.85 | 0.77 | 0.73 | 0.79 | 0.83 |
| AU-PRC | 0.88 | 0.88 | 0.84 | 0.88 | 0.80 | 0.88 |
| accuracy | 0.82 | 0.81 | 0.68 | 0.80 | 0.80 | 0.80 |
| precision | 0.81 | 0.80 | 0.69 | 0.76 | 0.76 | 0.79 |
| recall | 0.93 | 0.93 | 0.89 | 0.98 | 0.99 | 0.94 |
| F1 score | 0.87 | 0.86 | 0.78 | 0.86 | 0.86 | 0.86 |
| MCC | 0.61 | 0.59 | 0.27 | 0.57 | 0.59 | 0.57 |
| SPC | 0.64 | 0.62 | 0.33 | 0.48 | 0.47 | 0.57 |
| NPV | 0.84 | 0.83 | 0.64 | 0.93 | 0.98 | 0.85 |

AU-ROC, area under the receiver operating characteristic curve; AU-PRC, area under the precision-recall curve; MCC, Matthews correlation coefficient; SPC, specificity; NPV, negative prognostic value; KNN, k-nearest neighbors classifier; LR, logistic regression; SVM, support vector machine; DT, decision tree; MLP, multilayer perceptron.
